# Characterization of Gene Expression Signatures for the Identification of Cellular Heterogeneity in the Developing Mammary Gland

**DOI:** 10.1007/s10911-021-09486-3

**Published:** 2021-05-14

**Authors:** Samantha Henry, Marygrace C. Trousdell, Samantha L. Cyrill, Yixin Zhao, Mary. J. Feigman, Julia M. Bouhuis, Dominik A. Aylard, Adam Siepel, Camila O. dos Santos

**Affiliations:** 1 Cold Spring Harbor Laboratory, Cold Spring Harbor, NY, 11724 US; 2 Graduate Program in Genetics, Stony Brook University, NY, 11794 US; 3 Erasmus University Medical Center, Rotterdam, Netherlands; 4 College of Biological Sciences, University of California, Davis, CA 95616 US

**Keywords:** Single-cell RNA sequencing, Mammary epithelial lineages, Mammary immune cells, Gene expression, breast epithelial evolution

## Abstract

The developing mammary gland depends on several transcription-dependent networks to define cellular identities and differentiation trajectories. Recent technological advancements that allow for single-cell profiling of gene expression have provided an initial picture into the epithelial cellular heterogeneity across the diverse stages of gland maturation. Still, a deeper dive into expanded molecular signatures would improve our understanding of the diversity of mammary epithelial and non-epithelial cellular populations across different tissue developmental stages, mouse strains and mammalian species. Here, we combined differential mammary gland fractionation approaches and transcriptional profiles obtained from FACS-isolated mammary cells to improve our definitions of mammary-resident, cellular identities at the single-cell level. Our approach yielded a series of expression signatures that illustrate the heterogeneity of mammary epithelial cells, specifically those of the luminal fate, and uncovered transcriptional changes to their lineage-defined, cellular states that are induced during gland development. Our analysis also provided molecular signatures that identified non-epithelial mammary cells, including adipocytes, fibroblasts and rare immune cells. Lastly, we extended our study to elucidate expression signatures of human, breast-resident cells, a strategy that allowed for the cross-species comparison of mammary epithelial identities. Collectively, our approach improved the existing signatures of normal mammary epithelial cells, as well as elucidated the diversity of non-epithelial cells in murine and human breast tissue. Our study provides a useful resource for future studies that use single-cell molecular profiling strategies to understand normal and malignant breast development.

**Supplementary Information:**

The online version contains supplementary material available at 10.1007/s10911-021-09486-3.

## Introduction

The heterogenous cellularity of solid tissues controls the specialized events needed for prompt transitions through various stages of development and tissue function. Particularly for mammary tissue, a comprehensive understanding of the developing gland begins with the ability to appreciate how its constituent parts coexist and maintain tissue homeostasis and milk production. Within the mammary epithelium, immature, stem-like cells (Mammary Stem Cells or MaSCs) support repopulation of the myoepithelial and luminal cell lineages [1–5]. Myoepithelial cells, that are connected to the basement membrane, further interact with luminal cells to aid in the contraction of the mammary ducts in response to offspring suckling [6, 7]. Luminal cells comprise an array of distinct cellular states, which drive processes associated with milk production [8, 9]. Mammary fibroblasts, which reside in proximity to myoepithelial cells, contribute to branching expansion and epithelium survival [10, 11]. Similarly, immune cells play a role in branching morphogenesis of the mammary epithelium and tissue regression during post-lactational involution [12, 13]. These diverse cell types sustain the plasticity of the mammary gland through successive stages of puberty, gestation, lactation and involution, making it one of the most developmentally dynamic tissues in mammals.

Generally, transcriptional regulation represents one of the key mechanisms that drive mammary epithelial cell plasticity. To extrapolate information from underlying transcriptional networks, previous studies have employed several strategies to link cellular and molecular states to mammary epithelial identity. For example, combining flow cytometric isolation with functional cellular markers has improved our understanding of the dynamics of lineage commitment, differentiation processes and mammary tissue development [5, 14–17]. More recently, single-cell sequencing strategies have enabled the interpretation of contiguous cellular cues and epithelial lineage dynamics in the developing mammary gland [8, 15, 18–23].

In this study, we utilized differential tissue fractionation and single cell RNA sequencing (scRNA-seq), to expand molecular signatures that assign lineage identity, and to characterize the heterogeneity of epithelial, immune and stromal cells within the post-pubescent murine mammary gland. This strategy enabled the examination of molecular signatures for both mammary epithelial and non-epithelial cell populations, which have remained unresolved in prior approaches. Our analysis also extends to datasets derived from women’s breast tissue, which allowed for the elaboration of gene signatures that resolved breast resident cell populations. By employing a single-cell, lineage identification approach, we further illustrate the evolutionary conservation of epithelial lineages across distant mammalian species through the comparative integration of analyses from human and murine mammary tissue. Collectively, our study provides a comprehensive gene signature for the characterization of mammary resident cell lineages, serving as a reference to better understand all aspects of cellular dynamics and evolutionary conservation during mammary gland development.

## Results

### Defining Mammary Epithelial and Non-epithelial Cell Populations

**Fig. 1:**
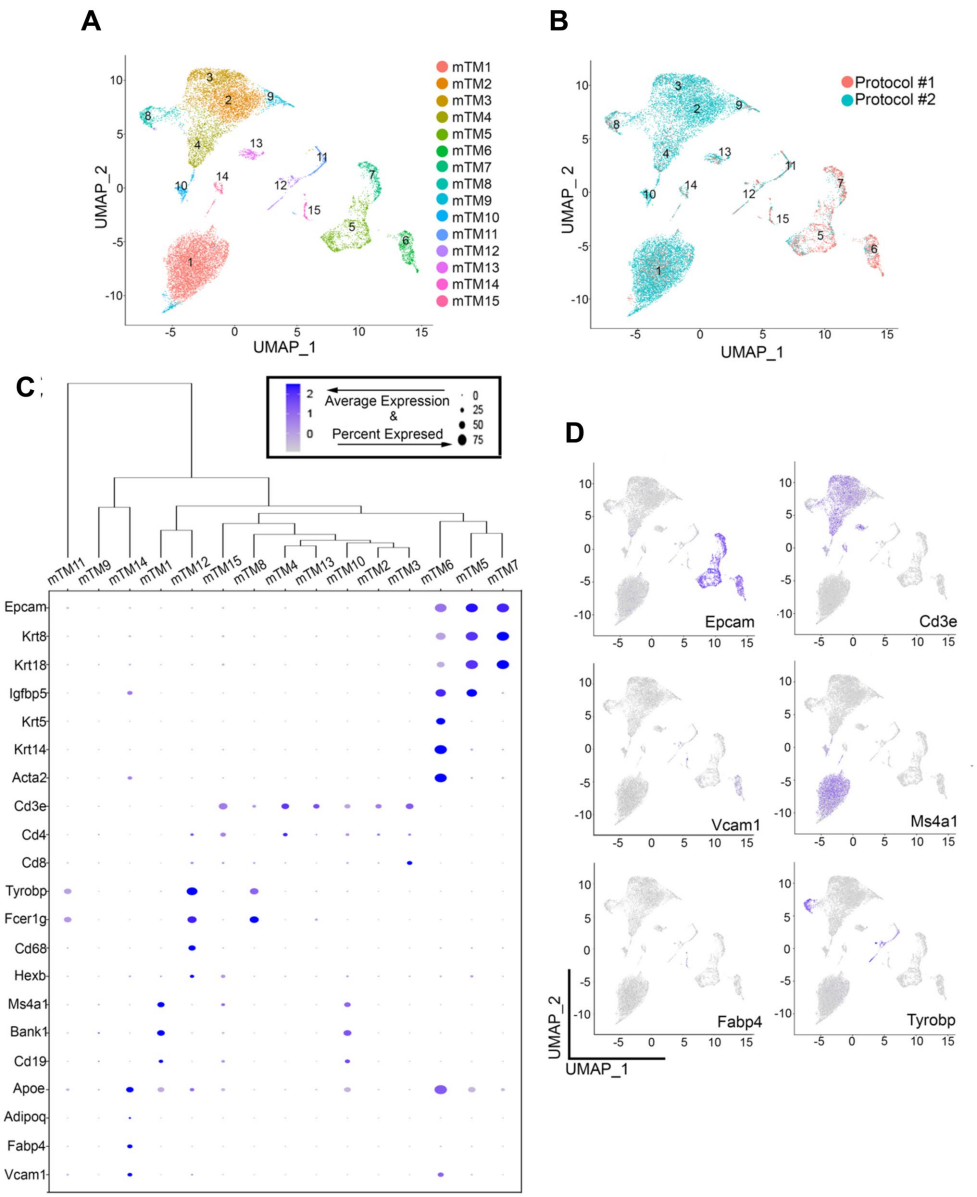
**Identification of specific populations of mammary epithelial and non-epithelial cells**. (**A**) UMAP plot showing murine Total Mammary clusters (mTM). (**B**) UMAP plot of cells showing the distribution of cells yielded from Protocol #1 and Protocol #2. (**C**) Dot plot and clustering (dendrogram) of mTM clusters shows the average and percentage of expressed genes that support classifying clusters, which include epithelial, lymphocytes, fibroblast and adipocyte-like cells. (**D**) Feature UMAP plots showing expression levels of epithelial markers (Epcam, Krt5, Krt18) and non-epithelial markers (Ms4a1, Cd3e and Tyrobp) across mTM clusters

To provide a comprehensive, molecular signature that allows for the resolution of population heterogeneity within the murine mammary gland, we employed two tissue dissociation protocols to selectively enrich for either epithelial cells (luminal and myoepithelial cells, Protocol #1) and non-epithelial cells (immune and stromal cells, Protocol #2) from mammary glands of adult, never pregnant, female mice (Supplementary Fig. S1A). scRNA-seq was performed on each of the enriched cells, and clustering analyses resolved fifteen total murine mammary clusters of cells (mTM), which were composed of a total of 15,359 cells isolated from both digestion strategies (Fig. 1A, B).

To differentiate between the epithelial and non-epithelial cell types within the fifteen mTM clusters, we utilized the transcriptional levels of classic markers of lineage identity. Clusters mTM5, mTM6 and mTM7 were comprised of epithelial cells given the expression of epithelial markers, such as Epithelial cell adhesion molecule (Epcam, pan-epithelial marker, mTM5, mTM6 and mTM7) [24], Cytokeratin 18 (Krt18, luminal epithelial marker, mTM7 and mTM5) [25, 26] and, Cytokeratin 5 (Krt5, myoepithelial/progenitor marker, mTM6) [26] (Fig. 1C, D). Immune cell lineages were classified according to the expression of Cluster of differentiation 3e (Cd3e, T-lymphocytes, mTM2, mTM3, mTM4, mTM13, mTM15) [27], Membrane Spanning 4-Domains A1 (Ms4a1, B-lymphocytes, mTM1, mTM9, mTM10) [28] and, Transmembrane immune signaling adaptor (Tyrobp, Myeloid, mTM8, mTM11, mTM12) [29]. Our analysis also indentified the presence of mixed-lineage stromal cells, marked by the expression of Actin alpha 2 (Acta2), and Fatty acid-binding protein 4 (Fabp4, putative fibroblasts and/or adipocyte-like cells, mTM14) [30–32] (Fig. 1C, D).

Such cluster identity classification indicated that utilization of Protocol #1 allowed for a 2.5-fold enrichment of murine mammary epithelial cells (mEC, luminal and myoepithelial cells) over Protocol #2, which yielded an 8-fold enrichment in murine non-epithelial cells (mNEC, fibroblasts, immune cells, adipocytes) (Supplementary Fig. S1B). Further gene expression analysis identified a series of genes, previously described to define specific lineage states of mammary epithelial populations, to be also expressed by cells from non-epithelial clusters [18, 21, 33, 34] (Supplementary Fig. S1C). Collectively, these analyses illustrated the technical relevance of tissue dissociation strategies for the characterization of cell-specific identities and analysis of cellular heterogeneity within mammary tissue.

## Improving the Classification of Mammary Epithelial Cell Populations

**Fig. 2:**
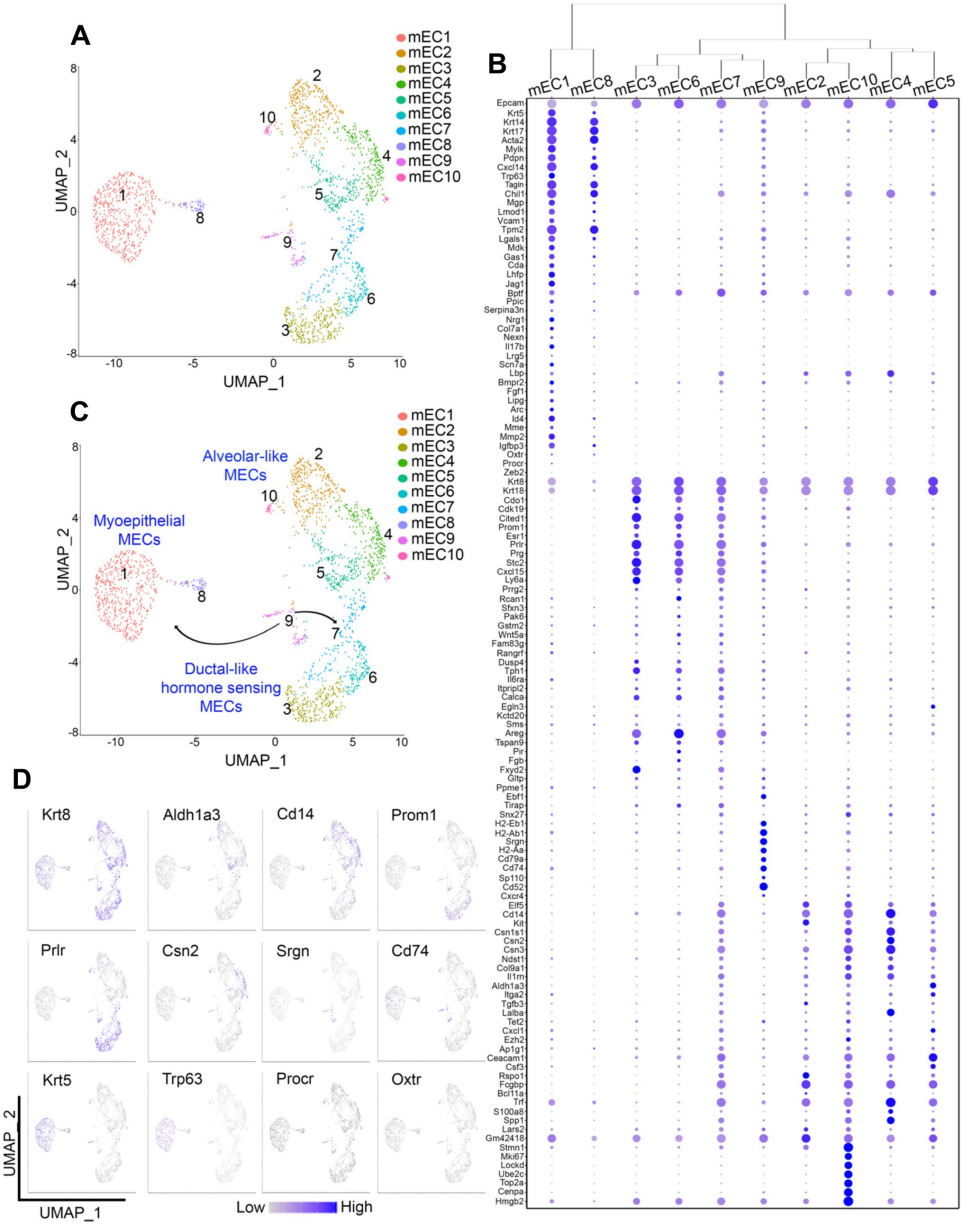
**RNA-seq profiles of FACS-isolated cells improve the identification of mammary epithelial cell populations**. (**A**) UMAP plot demonstrating distinct mECs from a re-cluster of cells with high mRNA expression levels for Epcam, Krt8, Krt18, Krt5 and Krt14. (**B**) Dot plot and clustering (dendrogram) of mEC clusters shows the average and percentage of expressed genes that support specific lineage cell type identification. (**C**) UMAP projection showing a monocle-informed transcriptional trajectory arrangement of mEC clusters. The arrows serve to suggest that cells tending toward a bipotential progenitor fate (mEC9) give rise to myoepithelial progenitors (mEC1) and predicted luminal common progenitors (mEC7). (**D**) Feature UMAP plots showing expression levels of specific genes in mEC clusters

To broaden the expression signatures that define epithelial lineage identities, we utilized a re-clustering strategy of the 2,016 cells expressing Epcam, Krt8, Krt18, Krt5 and Krt14. These secondary clusters comprised of epithelial cells arose from both digestion protocols but was predominantly composed of Protocol #1 cells (Supplementary Fig. S2A, B). Analysis of the expression of genes from distinct stages of the estrous cycle and cell cycle progression suggested similar cycle stages for epithelial cells from both protocols (Supplementary Fig. S2C, D). This re-clustering strategy yielded ten murine Epithelial Clusters (mEC), two of which corresponded to basal compartment cells (Krt5 + , mEC1 and mEC8) and eight corresponding to luminal cells (Krt18 + , mEC2-mEC7, mEC9 and mEC10) (Fig. 2A-B).

To define the distinct population of cells within the basal and luminal mammary compartments, we utilized previously published bulk RNA-seq datasets generated from FACS-isolated mammary epithelial cells (MECs) [14–17, 21, 33]. Using this strategy, we defined differentially expressed genes (DEGs) in MaSCs, myoepithelial progenitor cells, myoepithelial differentiated cells, luminal progenitor cells, luminal ductal cells, and luminal alveolar cells (Supplementary File S1). The analysis was then supplemented with DEGs defined for each of the scRNA-seq epithelial clusters, which together provided a comprehensive expression signature for the characterization of epithelial lineage identities (Supplementary Fig. S3A and Supplementary File S2).

In line with previous studies, myoepithelial cells were defined by the expression of basal compartment-biased genes Krt5, Cytokeratin 14 (Krt14), Cytokeratin 17 (Krt17), Acta2, Secreted Protein Acidic and Cysteine Rich (Sparc), Myosin light chain kinase (Mylk), Podoplanin (Pdpn) and C-X-C Motif Chemokine Ligand (Cxcl14) [15, 22, 26, 34–36]. Myoepithelial progenitor/stem cells were marked by the expression of genes previously shown to contribute to tissue reconstruction in fat pad transplantation assays and overall mammary developmental processes, such as Tumor protein p63 (Tp63) [37], Bromodomain PHD Finger Transcription Factor (Bptf) [17], and classical markers of mammary stem-like state, such as Leucine Rich Repeat Containing G Protein-Coupled Receptor 5 (Lgr5) [38], Neuregulin 1 (Nrg1) [39] and Inhibitor of DNA Binding 4, HLH Protein (Id4) [40] (Fig. 2B and Supplementary Fig. S3A). Interestingly, we did not detect abundant levels of a few known markers of the MaSC-like state such as Protein C Receptor (Procr) [41], BAF chromatin remodeling complex subunit (Bcl11a) [42], and Zinc Finger E-Box Binding Homeobox (Zeb2) [41], across all epithelial cell clusters. The low expression of these genes could represent differences in mammary tissue processing, mouse strain, mouse age, and mouse estrous cycle stage between our dataset and previously published ones.

Cells from cluster mEC8, which demonstrated low levels of expression of the stem-like signature, were characterized as differentiated myoepithelial cells. A third cluster of cells, mEC9, originally defined as belonging to a Krt8 + Krt18 + luminal fate, expressed elevated levels of Krt5, and may represent a population of putative bipotential progenitors predicted to express markers from both lineages [15, 43] (Fig. 2B and Supplementary Fig. S3A). Furthermore, cells from the mEC9 cluster showed expression of a set of genes identified in MaSC-like cells such as Campath-1 antigen (CD52) [44], Ebf Transcription Factor 1 (Ebf1) [45], B-cell antigen receptor complex-associated protein alpha chain (CD79a) [14], HLA class II histocompatibility antigen gamma chain (CD74) [14], H-2 class II histocompatibility antigen, I-E beta chain (H2-eb1), H-2 class II histocompatibility antigen, A beta chain (H2-ab1), and H-2 class II histocompatibility antigen, A-B alpha chain (H2-Aa) [21]. These results suggest the presence of a small population of cells with a bipotential, luminal/basal molecular signature within the mammary gland (Fig. 2B and Supplementary Fig. S3A).

The analysis of cells predicted to belong to the luminal cell fate resolved an array of progenitor and differentiated cells, according to the expression of Cyclin Dependent Kinase 19 (Cdk19) [14], Cbp/P300 Interacting Transactivator with Glu/Asp Rich Carboxy-Terminal Domain 1 (Cited1) [8], and Cysteine Dioxygenase Type 1 (Cdo1) [46] (Fig. 2B and Supplementary Fig. S3A). Within these clusters, mEC3, mEC6, and mEC7 aligned with the functional classification of ductal-like cells, given the expression of the functional marker, Prominin-1 (Prom1/CD133) [47], and the concomitant expression of hormone responsive genes, such as Estrogen receptor 1 (Esr1), Progesterone Receptor (Pgr), and Prolactin Receptor (Prlr) [8] (Fig. 2B and Supplementary Fig. S3A). Further analysis of ductal-like cells from cluster mEC3 revealed distinct cluster markers, such as the expression of the gene FXYD Domain Containing Ion Transport Regulator 2 (Fxyd2), which participates in mammary expansion in response to pregnancy hormones [48], as well as other genes with unknown functions in mammary epithelial cells, including Protein Phosphatase Methylesterase 1 (Ppme1), the Glycolipid transfer protein (Gltp), Glutathione S-transferase Mu 2 (Gstm2), and the Inositol 1,4,5-triphosphate receptor interacting protein-like 1 (Itpripl1). We also found that the luminal ductal-like cells from cluster mEC6 abundantly expressed levels of additional genes not previously linked to mammary tissue such as Tetraspanin 9 (Tspan9) and Pirin (Pir).

In addition to Prom 1+ epithelial cells, our analysis identified populations of luminal alveolar-like cells (mEC2, mEC4, mEC5 and mEC10), that express casein-like genes, a cellular state that precedes the pregnancy-induced secretory alveolar fates [8, 21]. Interestingly, all clusters classified as alveolar-like cells expressed abundant levels of genes previously proposed to define a progenitor-like state, such as E74 Like ETS Transcription Factor 5 (Elf5), Monocyte differentiation antigen 14 (Cd14), KIT Proto-Oncogene, Receptor Tyrosine Kinase (Kit) and Enhancer Of Zeste 2 Polycomb Repressive Complex 2 Subunit (Ezh2), [8, 18, 21, 49, 50]. These results suggest an accumulation of partially differentiated luminal secretory cells in mammary tissue from a never pregnant, post-pubescent female mouse. We found that mEC4 cells expressed higher levels of Lactalbumin Alpha (Lalba) and S100 Calcium Binding Protein A8 (S100a8) mRNAs [8, 51], while those from mEC5 expressed high levels of Aldehyde Dehydrogenase 1 Family Member A3 (Aldh1a3) and Transferrin (Trf) mRNAs, all previously associated with luminal progenitor cells [27] (Fig. 2B and Supplementary Fig. S3A). Cells from mEC2 were marked by the expression of R-Spondin 1 (Rspo1) and Fc Fragment of IgG Binding Protein (Fcgbp) [52, 53], while cells from mEC10 expressed Stathmin 1 (Stmn1) and C-X-C Motif Chemokine Receptor 4 (Cxcr4) [54, 55], together suggesting the presence of multiple, alveolar-like progenitor states. Notably, cells from cluster mEC10 also expressed a set of genes associated with cellular growth and cell cycle progression, suggesting they exist in a state of cellular proliferation (Fig. 2B and Supplementary Fig. S2B).

Our MEC population analysis also identified a unique population of cells, cluster mEC7, marked by the expression of luminal progenitor-associated genes, such as Elf5, Cd14, Kit and Ezh2, as well as expression of both ductal-like hormone sensing markers (Prom1, Esr1, Pgr) and alveolar-like secretory markers Casein Beta (Csn2), Casein kappa (Csn3), Carbonic anhydrase 2 (Car2), and Bifunctional heparan sulfate N-deacetylase/N-sulfotransferase 1 (Ndst1) [56]. Further, cells in mEC7 selectively expressed the gene Tet Methylcytosine Dioxygenase 2 (Tet2), a DNA demethylase that plays a fundamental role in controlling the differentiation potential of luminal progenitor cells [57] (Fig. 2B and Supplementary Fig. S3A). Therefore, we propose the cells in mEC7 represent a common, luminal progenitor state that may give rise to both hormone sensing and secretory luminal cell subtypes, as has been previously suggested [8].

Given the array of putative progenitor cells defined by the expansion of lineage-associated expression signatures, we next asked whether we could predict cellular transitions using the transcriptomic profile of each cell cluster. General transcriptional trajectory analysis confirmed our original hypothesis, that cells from cluster mEC9 likely represent a bipotential luminal/basal progenitor, which may precede the myoepithelial progenitor (mEC1) and predicted luminal common progenitor cells (mEC7) in the MEC lineage tree. The defined transcriptional trajectories also suggested that cells from mEC7 further split into two luminal branches which, in turn, gave rise to ductal-like and alveolar-like cells (Fig. 2C,2D and Supplementary Fig. S3B).

Pathway analysis of predicted bipotent progenitor (mEC9) indicated an enrichment for genes that are associated with pathways that control mammary stem cell maintenance, such as Notch signaling and IL5-signaling [58, 59] (Supplementary Fig. S3C, pink and Supplementary File 3). Interestingly, both pathways have also been described to influence mammary lineage commitment, with Notch biasing commitment towards the myoepithelial fate, and IL-5 signaling driving luminal specification [58, 59]. Moreover, we found that both common luminal progenitors (mEC7) and luminal hormone-negative progenitors (mEC2), were enriched for genes associated with Wnt signaling, which has been previously implicated to coordinate proliferation and differentiation of mammary progenitor cells [60, 61] (Supplementary Fig.S3C, blue and ochre, Supplementary File 3). More specifically, common luminal progenitors (mEC7) were defined by genes associated with ErbB and Insulin signaling pathways, which have been linked with the maintenance and expansion of the luminal epithelial compartment [62, 63] (Supplementary Fig. S3C, blue, Supplementary File 3). Altogether, analyses of genes preferentially expressed in clusters mEC2, mEC7 and mEC9, identified by our scRNA-seq analysis, support their existence in a more undifferentiated state. Additionally, flow cytometric analysis validated several of these markers confirming our predictions of cellular state, lending further support to our approach in expanding molecular signatures that assign lineage identities (Supplementary Fig. S4). Collectively, these analyses revealed a complex balance of immature and differentiated mammary resident epithelial cells and their putative relevance in maintaining homeostasis in post-pubescent, mammary tissue.

## An Extended Molecular Signature Reveals Cellular Dynamics During Pregnancy-induced Mammary Gland Development

We next asked whether our extended molecular signatures could expand our understanding of cellular dynamics during mammary developmental processes. In doing so, we investigated a previously published dataset of scRNA-seq profiles derived from Epcam + mammary epithelial cells harvested from mice during mid gestation (day 14.5), early lactation (day 6) and late involution (11 days post-weaning) [8]. Given that these datasets were generated utilizing mammary cells from C57BL/6 female mice, we first investigated whether our ability to predict mammary lineages could be impacted by strain specific changes to gene expression [64–68]. We found that genes previously predicted to be differentially expressed between C57BL/6 and Balb/c show relative similar mRNA abundance in MECs from either dataset, which suggested that strain-specific variation in gene expression were not majorly represented in mammary tissue. We also identified a few genes that are more abundant in C57BL/6 nulliparous MECs, and those more abundant in Balb/c nulliparous MECs, and therefore consistent with the idea of strain-specific changes to a subset of gene expression (Supplementary Fig. S5A). Most importantly, genes defined to characterize epithelial lineage and identity showed no difference in abundance across MECs from both mouse strains, thus suggesting that a molecular definition of the epithelial cell compartment is unlikely to be influenced by strain-specific alterations to gene expression (Supplementary Fig. S5B).

**Fig. 3:**
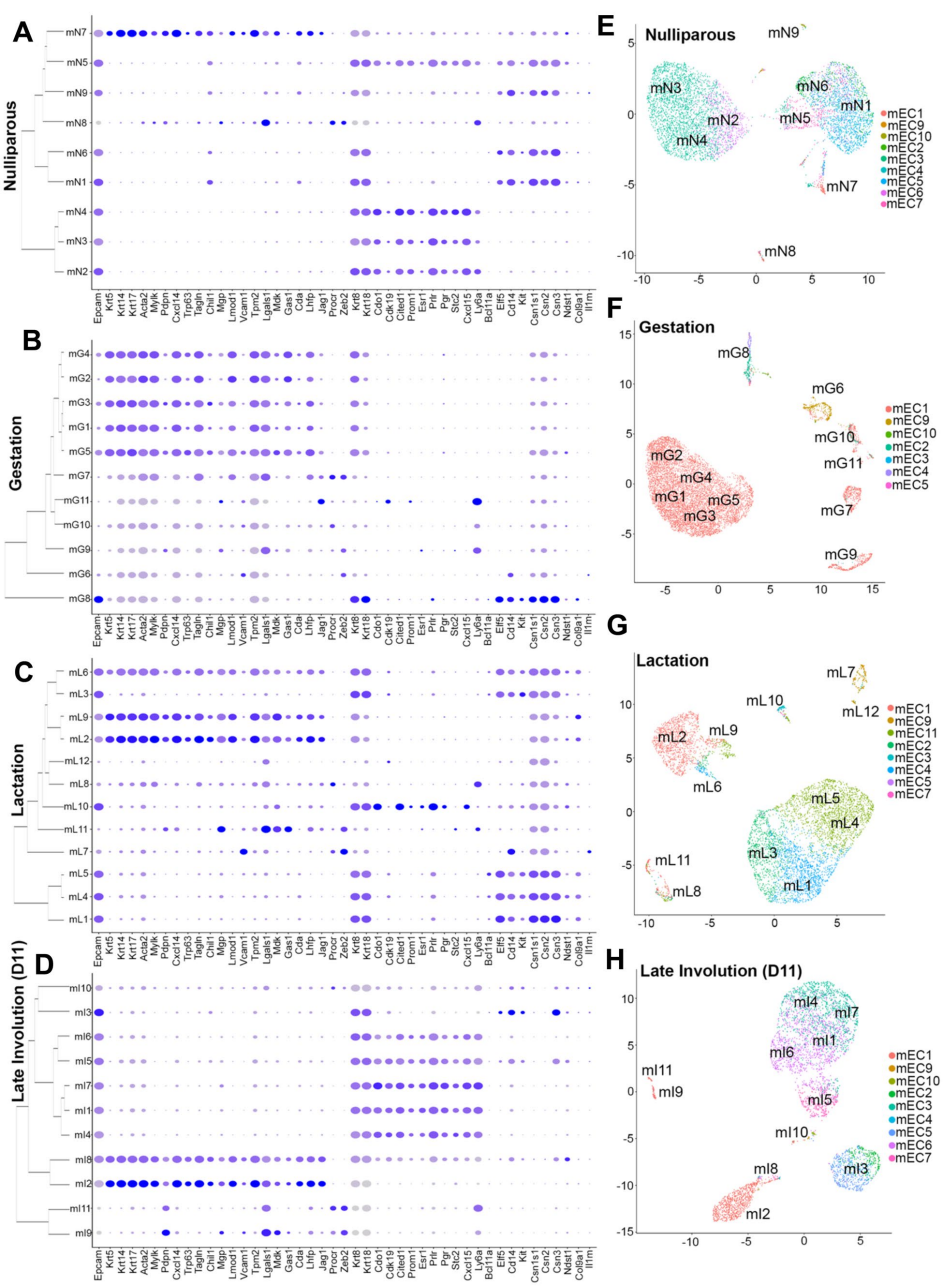
**Extended molecular signatures reveal cellular dynamics during pregnancy- induced mammary gland development.** (**A-D**) Dot plot and dendrogram branching showing average and percentage expression of epithelial genes utilized to characterize specific epithelial cell-type populations from mammary tissue harvested from (**A**) nulliparous female mice (mN) (**B**) female mice at mid-gestation (mG) (**C**) female mice during lactation (mL) and (**D**) female mice at late states of involution (mI). (**E–H**) UMAP displaying predicted cluster classifications of cells harvest from (**E**) nulliparous female mice (mN), (**F**) female mice at mid-gestation (mG), (**G**) female mice during lactation (mL) and (**H**) female mice at late states of involution (mI), in relation to clusters defined in mammary glands from nulliparous, post-pubescent female mice (mEC)

We next resolved a series of cellular clusters for each of the previously published mammary developmental stages datasets (Supplementary Fig. S6A, B, C and D). Each cell cluster was then analyzed according to the gene signatures defined in our scRNA-seq data from the nulliparous mammary cells (Fig. 2). Overall, this analysis validated the presence of cell types identified with our expanded gene signature in datasets derived from nulliparous mammary glands (mN clusters, Fig. 3A), and mammary tissue at other developmental stages (Fig. 3B, C, and D**)**.

We found that all eleven clusters identified during gestation (mG) were characterized by the expression of genes that define myoepithelial-like cells, such as Krt5, Krt14, Krt17, Acta2, amongst others, suggesting a mixed luminal-basal molecular identity (Fig. 3B). This was further supported by the analysis of luminal alveolar-like genes, such as Csn2, and Csn3 across all clusters. From the eleven clusters, mG1, mG2, mG3, mG4 and mG5 expressed higher levels of myoepithelial-biased markers, suggesting a more defined, myoepithelial state compared to other clusters. Conversely, cells from cluster mG8 had a molecular signature of the luminal, alveolar, progenitor state, given the high levels of Krt8, Krt18, Kit, Cd14 and Elf5 mRNAs. All clusters, apart from cluster mG8, expressed much lower levels of Epcam, Krt8 and Krt18 mRNAs, suggesting that signals during gestation may alter the expression of canonical markers of mammary epithelial lineage identification (Fig. 3B).

Our approach also investigated lineage-associated, molecular signatures in MECs isolated from mice during lactation (Fig. 3C). All epithelial clusters identified in lactating glands (mL) expressed high levels of milk-associated proteins, Casein Alpha S1 (Csn1s1), Csn2 and Csn3, with clusters mL1, mL3, mL4, mL5 and mL6 expressing markers of progenitor, alveolar- fate such as Kit, Cd14 and Elf5. These clusters also expressed moderated levels of Prolactin receptor (Prlr), thus supporting a milk-sensing, cellular state. Interestingly, cluster mL6 was marked by the expression of myoepithelial-like and luminal-like genes, suggesting the presence of an epithelial bipotential cellular state during lactation. Cluster mL10 uniquely expressed luminal ductal-like genes and higher levels of genes encoding the hormone receptors, Esr1 and Pgr, in contrast to clusters mL2 and mL9, which presented a myoepithelial-biased gene signature (Fig. 3C).

Clusters identified during the late stages of involution (mI) display a more lineage-defined state with clearer distinction between myoepithelial-like cells (clusters mI2 and mI8), and luminal-like cells (mI1, mI3, mI4, mI5, mI6, mI7, mI9, mI10 and mI11). Among the clusters with luminal-biased signatures, we observed a greater representation of luminal, ductal-like cell populations (clusters mI1, mI4, mI5, mI6, mI7 and mI8), with some of them carrying both, alveolar-like and ductal-like signatures (mI5 and mI8). In fact, identification of a single cluster of cells with definitive expression of alveolar-like, casein genes (mI3) support the transition from a predominantly secretory state during lactation into a non-secretory, homeostatic state. Moreover, three clusters (mI9, mI10 and mI11) comprised of myoepithelial-like and luminal-like genes support the activation of a variety of stem-like cells during involution for tissue reconstruction after pregnancy (Fig. 3D).

We next decided to utilize broader prediction models to investigate, without bias, whether global expression patterns from post-pubescent mammary epithelial cells (mEC, Fig. 2) could infer the cellular state of epithelial cells during gestation, lactation and involution. Importantly, utilizing this approach would support the effectiveness of our extended molecular signatures in defining lineage identities across stages of adult mammary development. In concordance with our previous findings, MECs isolated from C57BL/6 nulliparous female (indicated as mN clusters) mice fit within the population distribution presented in Fig. 2, except for a population characterized as mature myoepithelial cells (mEC8) which was not present in any of the datasets generated from C57BL/6 animals (Fig. 3E, F, G and H). This difference could be explained by either the marker-specific isolation used for the C57BL/6 MECs (Epcam), or the relatively small abundance of cells within the mEC8 cluster during pregnancy-induced development.

Furthermore, we confirmed that cells isolated during gestation displayed a myoepithelial-biased molecular identity, given that many of the gestation cell clusters (mG1, mG2, mG3, mG4, mG5, mG6, mG8, mG9, and mG10), show gene expression patterns that resemble those defined in myoepithelial progenitor/stem-like cells (Fig. 3F). Analyses of lactation-derived cellular clusters also confirmed our gene signature analyses, showing that global patterns of gene expression associated with several clusters (mL1, mL4, mL5, mL6, mL9) of luminal, alveolar-like cell fates in the pre-pubescent mammary glands (Fig. 3G). As such, predictions of global transcription suggested the prevalence of cells with a ductal-biased state during involution (Fig. 3H), thus supporting a complex cellular state during gestation, lactation and involution. Our analyses also demonstrated that populations of MECs classified as bipotent (mEC9), were present across all developmental stages analyzed, whereas clusters with a more luminal, immature signatures (mEC2 and mEC7) were more abundantly detected in nulliparous tissue, and during lactation and involution.

Pathway analyses illustrated differentially enriched networks within each of these clusters across pregnancy-induced mammary development (Supplementary File S4). Overall, C57BL/6 nulliparous predicted bipotent (mN9) and progenitors (mN6 and mN5), were enriched for similar pathways to those of Balb/c MECs, thus supporting that mechanisms that control mammary cellular states are maintained across distinct murine strains (Supplementary Fig. S3C and Supplementary Fig. S7). Throughout the pregnancy-induced development, predicted mammary bipotent progenitors (gestation cluster mG6, lactation clusters mL7 and late involution cluster mI10) were enriched for immune communication pathways, mechanisms that could represent an adaptive signal for protection of stem-like cells during development, in response to the immune suppression that accompanies pregnancy-induced development. During gestation and lactation, bipotent progenitors were marked by pathways associated with calcium regulation, cellular relaxation and contraction (mG6 and mL7), and IL-3 signaling (mL7), all of which are mechanisms regulated by increased levels of Prolactin and known to play an important role during pregnancy-induced mammary development [69]. At the end of involution, the enrichment of genes associated with apoptotic signaling, insulin response and adipogenesis suggest the presence of mechanisms, that are associated with hallmarks of post-pregnancy mammary involution [70, 71] (Supplementary Fig. S7).

Pathway analysis for the putative luminal hormone-negative MECs (gestation cluster mG8, lactation cluster mL3, and late involution cluster mI3) and common luminal progenitor MECs (lactation cluster mL10 and late involution cluster mI5) showed enrichment of a distinct set of gene networks at each of the pregnancy-induced, mammary gland developmental stages. During gestation, luminal hormone-negative MECs (mG8) were enriched for pathways associated with lipid metabolism, suggesting their initial steps towards milk production (Supplementary Fig. S7). Curiously, our cellular prediction analysis failed to identify cells with a common luminal progenitor signature during gestation (Fig. 3F). The lack of cells carrying this signature during gestation could either be indicative of the rapid rate of differentiation of luminal cells in response to pregnancy hormones or may represent the pan-cellular alteration of gene expression signatures as observed in other cell types during the same developmental stage (Fig. 3B).

Moreover, during lactation, both luminal hormone-negative MECs and common luminal progenitor MECs were marked by the enrichment of pathways associated with prostaglandin synthesis and regulation, which have been associated with lactogenic potential of MECs and milk maturation [72] (Supplementary Fig. S7). Both cell states were also enriched for genes associated with TNFα - NF-κβ signaling pathway, immune communication and adipogenesis, suggesting their role in mammary gland clearance and tissue remodeling post-lactation [70, 71] (Supplementary Fig. S7). Collectively, our ability to predict immature, cellular states during mammary gland development, via the expansion of molecular signatures of nulliparous MECs, have enabled the prediction of their specific functional roles in response to signals present during gestation, lactation and involution.

In addition to the specific, molecular programs expressed by MECs during the pregnancy cycle, those residing in the post-involuted mammary gland bear unique and stable molecular signatures [73–75]. Therefore, it is possible that these molecular changes could represent a combination of altered mammary cellular heterogeneity and differential transcriptional output of epithelial cells. With the intent to address this question, we next investigated whether the expression of genes previously described as a parity-induced signature [74], were exclusive to mammary epithelial cells, or shared across other mammary resident cell types (Fig. 1). We found that the majority of the parity-induced genes were expressed in our dataset, with the exception of the genes Secreted frizzled-related sequence protein 4 (Sfrp4), Trypsin-like serine protease (Sprx), Mast cell protease 2 (Mcpt2), Cop9 signalosome complex subunit 2 (Cops2), Cop9 signalosome complex subunit 7 (Csn7), Carboxylesterase (Ces) and Carbonic anhydrase 3 (Ca3). Further analysis indicated that approximately 60% of these parity-induced signature genes were expressed similarly in mammary epithelial and non-epithelial cells, with several genes being more abundantly expressed in non-epithelial cells (Supplementary Fig. S8A, B). Our results support the reliable use of this parity-associated gene signature to define the pregnancy state of pre-isolated MECs but raise the outstanding question of whether non-epithelial cell lineages and heterogeneity contribute significantly to the post-pregnancy state of mammary tissue. Taken together, our analyses support an extended molecular signature of mammary epithelial lineages to improve the identification of cellular dynamics, even in conditions where the response to pregnancy signals induces molecular and cellular alteration to the mammary gland.

## Outlining the Diversity of Non-epithelial Mammary Resident Cell Types

**Fig. 4:**
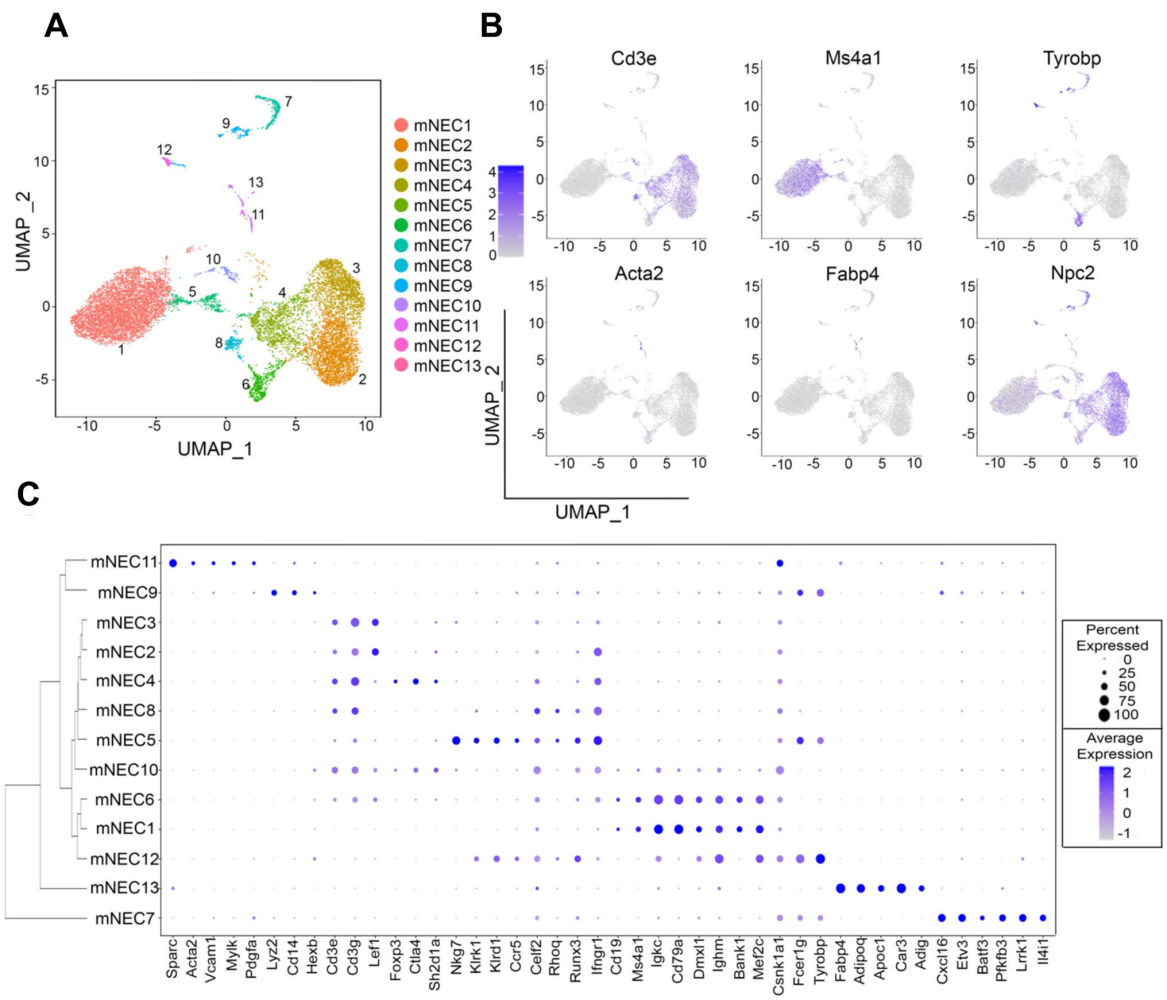
**Outlining the diversity of mammary resident non-epithelial cell types.** (**A**) UMAP plot demonstrating distinct murine Non-Epithelial cell clusters (mNEC) from a re-cluster of non-epithelial cells expressing low levels of Epcam, Krt18, Krt8, Krt5 and Krt14 mRNAs. (**B**) Feature UMAP plots show expression levels of Cd3e, Ms4a1, Tyrobp, Acta2, Fabp4 and Npc2 mRNAs in mNEC clusters. (**C**) Dot plot and dendrogram branching showing average and percentage of expressed genes used to distinguish and classify diverse immune cell populations

Given that immune and stromal cells play a central role during mammary gland development and tissue homeostasis [5], we next focused on defining the diversity of non-epithelial cells in post-pubescent never pregnant mammary tissue. We re-clustered non-epithelial cells (12,646 cells, with low mRNA levels for Epcam, Krt18, Krt8, Krt5 and Krt14 genes, Fig. 1C), an approach that yielded thirteen unique clusters of murine non-epithelial cells (mNEC, Fig. 4A). As we expected, we obtained most of the non-epithelial cells from tissue dissociation Protocol #2. (Supplementary Fig. S9A, B). Analyses of classic markers that define T-lymphocytes (Cd3), B-lymphocytes (Ms4a1), Myeloid cells (Tyrobp), Fibroblasts (Acta2), and Adipocytes (Fabp4) demonstrated the diversity of lineage identities of non-epithelial cells residing in mammary tissue (Fig. 4B).

With the goal of building molecular signatures to better define non-epithelial cellular identities, we investigated the top DEGs across all non-epithelial cell clusters (Supplementary File S5). Utilizing classical markers B-lymphocyte antigen Cluster of Differentiation 19 (Cd19) and Ms4a1 [76, 77], our analyses identified two clusters with B-lymphoid lineage identity (mNEC1 and mNEC6). They both showed the expression of immunoglobulin-like genes Immunoglobulin Kappa Constant (Igkc), Immunoglobulin Heavy Constant Mu (Ighm), Immunoglobulin Heavy Constant Delta (Ighd) and additional B-cell markers, such as Cd79a [78], Cluster of Differentiation 83 (Cd83) [79], B Cell Scaffold Protein with Ankyrin Repeats 1 (Bank1) [80], and Paired Box 5 (Pax5) [81] (Fig. 4C, and Supplementary Fig. S9D). Interestingly, B-cell populations clustered closely with Natural Killer (NK) cells (mNEC12), cells known for playing an important role during mammary gland involution and breast tumorigenesis [82, 83], and that are defined by genes like Killer Cell Lectin Like Receptor K1 (Klrk1) [84], Killer Cell Lectin Like Receptor D1 (Klrd1) [85], and Sialic acid binding Ig-like lectin H (Siglech) [86] (Fig. 4C, and Supplementary Fig. S9D).

Our analysis also identified a variety of T-lymphocytes, characterized by the expression of Cd3e mRNA. Among these, we identified Cluster of differentiation 4 (Cd4 + , mNEC2) and Cluster of differentiation 8 (Cd8 + mNEC3) expressing cells, which expressed high levels of T cell master regulator Lymphoid Enhancer Binding Factor 1 (Lef1) [87, 88] (Fig. 4C, Supplementary Fig. S9C, D). In addition, we identified clusters of Cd4 + regulatory T-like cells (Treg, mNEC4 and mNEC10), which were marked by the expression of Treg lineage factor Forkhead box P3 (Foxp3) [89] and other Treg associated genes such as Cytotoxic T-Lymphocyte Associated Protein 4 (Ctla4) [90] and SH2 Domain Containing 1A (Sh2d1a) [91] (Fig. 4C, Supplementary Fig. S9D). Interestingly, mNEC10 Tregs were exclusively marked by the expression of cell cycle control genes, such as DNA Topoisomerase II Alpha (Top2a), Marker of Proliferation Ki-67 (Mki67) and Ubiquitin Conjugating Enzyme E2 C (Ube2c), suggesting a proliferative cellular state (Fig. 4C, Supplementary Fig. S9D). Moreover, we identified two clusters of Cd4^–^Cd8^–^ NKT-like cells (mNEC5 and mNEC8), that expressed Cd3 mRNA and NK-associated genes such as Natural Killer Cell Granule Protein 7 (Nkg7) [92], Klrk1[84], and CUGBP Elav-Like Family Member 2 (Celf2) [93, 94], a population of cells not well explored in normal mammary tissue, but that has also been implicated during mammary tumorigenesis [95] (Fig. 4C, Supplementary Fig. S9D).

Outside of the lymphocytic-biased lineage, we identified myeloid-biased clusters, including a population of dendritic cells (mNEC7), marked by the expression of dendritic master regulator Basic Leucine Zipper ATF-Like Transcription Factor 3 (Batf3) [96], and macrophage-like cells characterized by the expression of classical markers such as Cd14 [97], Lysozyme C-2 (Lyz2) [98] and Hexosaminidase Subunit Beta (Hexb) [99] (Fig. 4C, Supplementary Fig. S9D). Additional myeloid populations (neutrophils, monocytes) were not detected in our datasets, perhaps due to their potential low abundance in post-pubescent murine mammary tissue. Fibroblasts (mNEC11) and Adipocyte-like cells (mNEC13) were also detected in our datasets, based on molecular signatures including Platelet Derived Growth Factor Subunit A (Pdgfa) and Adiponectin (Adipoq) respectively (Fig. 4C, Supplementary Fig. S9D). Collectively, our studies illustrate the effectiveness of short-term mammary digestion for the selective enrichment of mammary stromal and immune cells, including adipocytes and under-studied immune cell types. Our study also provided gene signatures and re-clustering strategies that will enable the efficient characterization of the diversity of such cell types in future scRNA-seq analyses of the developing mammary gland.

## Expanded Molecular Signature of Epithelial and Non-epithelial Human Breast Tissue

**Fig. 5:**
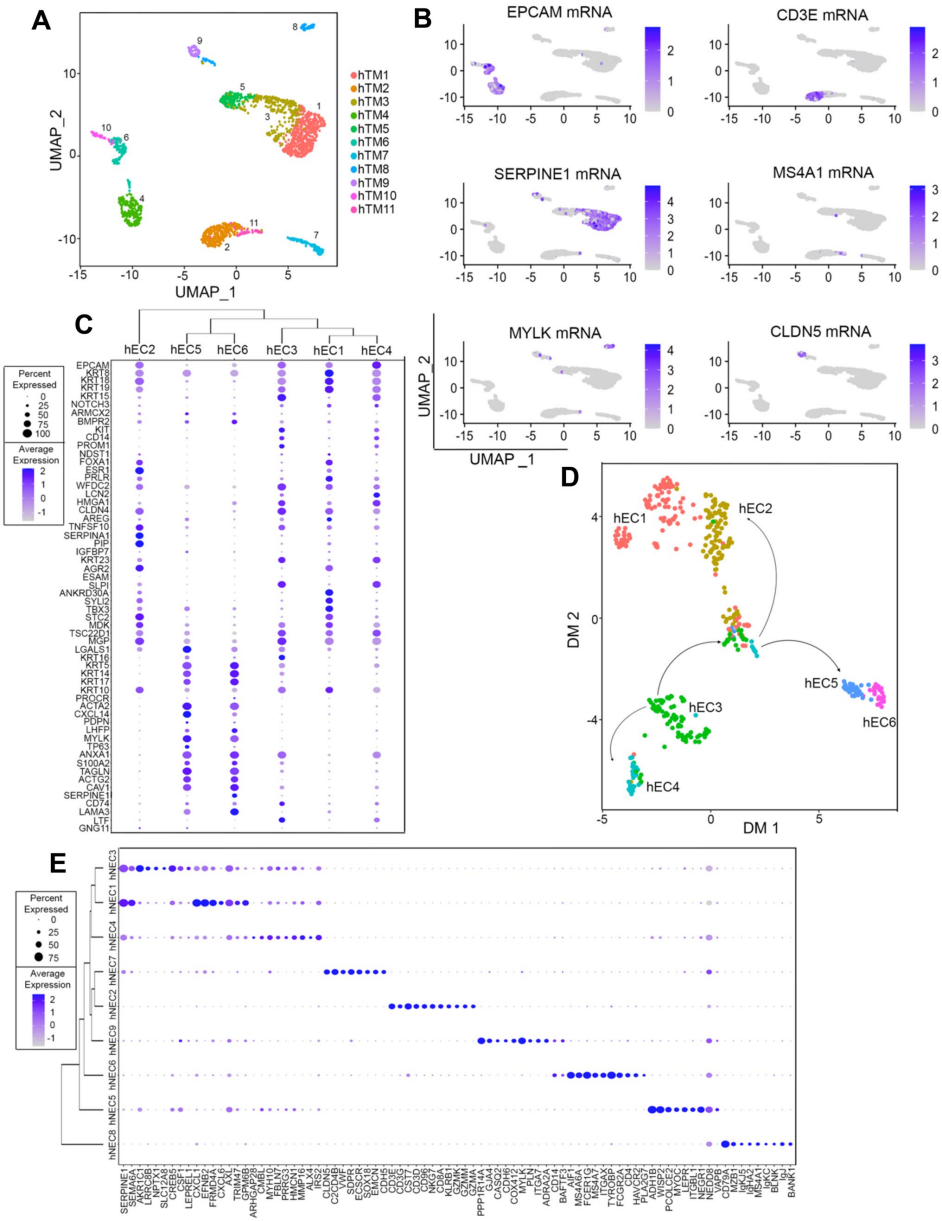
**Expanding the molecular signature of epithelial and non-epithelial human breast tissue.** (**A**) UMAP plot showing cluster distribution of non-cancerous, total breast tissue sample, from nulliparous women (n = 5, hTM). (**B**) Feature UMAP plots showing expression levels of EPCAM, SERPINE1, CD3E, MS4A1, CLDN5 and MYLK mRNAs in hTM clusters. (**C**) Dot plot and dendrogram branching showing the average and percentage of expressed genes that distinguished and classified clusters of epithelial cell lineages (hECs). (**D**) Diffusion map projection showing a Monocle-informed transcriptional trajectory arrangement of hEC clusters. The arrows serve to suggest that cells likely part of a bipotential progenitor fate (hEC3) give rise to myoepithelial progenitors (hEC5) and predicted luminal progenitors (hEC2 and hEC4). (**E**) Dot plot and clustering (dendrogram) of hNEC clusters shows the average and percentage of expressed genes that support classifying clusters, which include non-immune populations of breast resident cells like endothelial cells, fibroblasts and adipocytes

We next performed scRNA-seq analysis of total, non-cancerous, nulliparous, human breast tissue, with the goal to further expand a molecular signature that predicts the lineage identity of breast resident cells (Supplementary Fig. S10A). Analysis of five human breast samples yielded eleven clusters of total breast cells (hTM), which represented clusters of breast epithelial cells (EPCAM +), myeloid cells (SERPINE1 +), T- lymphocytes (CD3E +), B-lymphocytes (MS4A1 +), endothelial-like cells Claudin 5 (CLDN5) and fibroblast-like cells (MYLK +) (Fig. 5A-B, Supplementary Fig. S10B). In addition, cell cycle analysis of the hTM clusters suggested a similar cycle progression across all clusters, supporting that the cell clustering was likely based on overall gene expression rather than differential expression of genes associated with cell cycle (Supplementary Fig. S10C).

The employment of online tools, commonly utilized to assign cell identities based on the gene expression of each cluster, yielded different cellular predictions to all clusters with the exception of cluster hTM11, which was predicted to have a B-cell lineage identity across all platforms (Supplementary File S6 and Supplementary Fig. S10D). Further analysis utilizing classic lineage markers confirmed the B-cell identity of hTM11, in addition to predicting identities of epithelial clusters hTM4, hTM6, hTM8 and hTM10 (EPCAM + , KRT8 + and KRT5 +) and non-epithelial clusters, based on the expression of immune markers (CD3E, Granzyme A (GZMA), Serpin Family E Member 1 (SERPINE1), A Receptor Tyrosine Kinase (AXL) and HEXB, endothelial markers CLDN5 [13, 27, 77, 99–104], fibroblasts marker Myocilin (MYOC) [105], and adipocytes markers Gap Junction Protein Alpha 4 (GJA4), and Procollagen C-Endopeptidase Enhancer (PCOLCE) [106, 107]. We also noted that some lineage specific markers of non-epithelial cells were expressed by epithelial lineages, such as HEXB and AXL (Supplementary Fig. S10E). Taking together, our results illustrate the overall complexity of lineage composition of human breast tissue.

We next focused on defining the identity of the epithelial lineages in the human breast. Using a similar approach employed for the characterization of murine epithelial cells, we identified clusters hTM4, hTM6, hTM8, and hTM10 that expressed high levels of epithelial markers (EPCAM, KRT8, and KRT18, KRT5, and KRT14) and performed re-clustering analysis of these cell populations. We also detected cells with relatively low expression of marker KRT18 (cluster hTM9). Given the lack of additional markers (EPCAM, KRT8, KRT5), these were not included to the epithelial focused re-clustering (Supplementary Fig. S10E).

With such approach, we defined six epithelial clusters (hEC), which we further characterized to define breast epithelial lineage identities (Supplementary Fig. S11A, B). The combination of markers that defined mouse mammary lineages (Fig. 2), and top DEGs for each cluster, permitted the expansion of gene signatures that defined each of the epithelial clusters (Fig. 5C, Supplementary File S7, and Supplementary Fig. S11C). We defined clusters of luminal-fates (hEC1, hEC2, and hEC4), which were classified as luminal differentiated prolactin receptor high (PRLRh, hEC1), luminal differentiated estrogen receptor high (ESR1h, hEC2) and luminal progenitor prolactin receptor high (PRLRh, hEC4) cells, according to their expression of EPCAM, KRT8, KRT18, KRT19, Claudin 4 (CLDN4), ESR1, PRLR mRNAs (Fig. 5C and Supplementary Fig. S11C). Our analysis identified cells from the myoepithelial lineage (hEC5 and hEC6), which included myoepithelial progenitors (hEC5) and differentiated myoepithelial cells (hEC6), given the expression of KRT5, KRT14, Laminin Subunit Alpha 3 (LAMA3), ACTA2, TP63, and Oxytocin Receptor gene (OXTR). We also found that cluster hEC3, expressed both classical luminal cell markers (KRT8, KRT15) and myoepithelial cell markers (KRT5, KRT14, KRT16), in addition to progenitor markers CD14 and KIT, suggesting a putative immature, bipotent, cellular state (Fig. 5C and Supplementary Fig. S11C). Transcription trajectory predictions further suggested that cluster hEC3 may occupy an intermediary position during human mammary epithelial differentiation across luminal and myoepithelial identities (Fig. 5D and Supplementary Fig. S11D).

Further molecular analysis revealed specialized mechanisms associated with each of the epithelial identities (Supplementary Fig. S11E, F, G, H, I and J, and Supplementary File 8). Luminal identities were supported by the enrichment of pathways associated with Prolactin signaling in luminal differentiated PRLR cells (hEC1), while luminal differentiated ESR1 cells (hEC2) were enriched for both Prolactin and Estrogen Receptor signaling, in addition to other pathways associated with hormonal responses and tissue homeostasis. Cells from cluster hEC4, classified as luminal progenitor PRLR cells were marked by the enrichment of NRF2 and RANKL/RANK signaling pathways, which have been described to regulate the homeostasis and differentiation of luminal progenitor cells [108, 109]. Predicted epithelial bipotent breast cells (hEC3) were enriched for breast stem-associated signatures, such as PDGF pathway, which is known to control the proliferation of mesenchymal cells in the breast [110], and Signal transduction through IL1R [111], suggesting a stem-like phenotype of these cells. Cells classified as myoepithelial progenitors (hEC5) and myoepithelial differentiated breast cells (hEC6) shared pathways associated with Cell contractibility, a hallmark of such cellular fate. Moreover, cells from cluster hEC5 were enriched for genes associated with Androgen receptor signaling, and inhibition of this pathway has been shown to enhance the estrogen-induced, proliferation of breast epithelial cells [112].

We next investigated whether our expanded signature of human breast lineages would define the identity of epithelial cells from additional scRNA-seq studies. In doing so, we validated our approach on a dataset that integrated scRNA-seq of FACS-isolated, human breast epithelial cells and tissue spatial analysis to define heterogeneity among mammary populations (NgNC, sample Ind #4) [20]. With the utilization of prediction models, we found a substantial overlap across cellular distributions between both datasets. This result was obtained despite the reduced number of human breast epithelial cells present in our dataset, supporting that our methodology is compatible with low-input samples and is sufficient for delineating the overall epithelial diversity present in the human breast sample (Supplementary Fig. S12A). Interestingly, cluster hEC4, defined in our dataset to represent a small population of luminal progenitor cells was predicted to be absent in the Ind#4 dataset, suggesting a potential challenge in the identification of rare cell populations. However, given that the parity state of this sample was unknown, the absence of this population could also reflect intrinsic changes to breast tissue due to developmental variation (Supplementary Fig. S12B). Analyses of additional datasets derived from FACS-isolated, breast epithelial tissue from nulliparous women (NgNC, sample Ind #5, Ind #6 and Ind #7) [20] also demonstrated substantial overlap across cellular distributions with our datasets, thus supporting the overall representation of major breast cell lineages across independently generated scRNA-seq profiles (Supplementary Fig. S12C). Within this set of analyses, cluster hEC2 defined in our dataset to represent a population of luminal differentiated ESR1 cells were not detected across additional nulliparous samples (Supplementary Fig. S12D). These findings may suggest that in addition to parity state, tissue diversity across individuals, tissue dissociation approaches, or focused epithelial cell isolation could also influence the diversity of breast epithelial cells identified in scRNA-seq analysis.

Together, these results support that the expansion of gene signatures, that define lineage identities of breast resident cells, is required for understanding cellular dynamics and heterogeneity during tissue homeostasis. More importantly, taking the approach of defining baseline, cellular identities may also improve our understanding of developmentally-induced alterations, including those that support cancer development and progression.

Next, we defined the non-epithelial population of breast resident cells. For this analysis, hTM clusters with low or absent expression of epithelial markers (EPCAM, KRT8, KRT18 KRT5, and KRT14) were re-clustered, yielding nine clusters of human non-epithelial cells (hNEC), which were further classified based on markers that defined mouse mammary lineages (Fig. 4), and the top DEGs for each cluster (Supplementary Fig. S13A, B and Supplementary File S9).

This analysis identified three clusters with myeloid-like lineage identities (hNEC1, hNEC3, and hNEC4), which were further classified as neutrophil-like cells (hNEC1), macrophage-like cells (hNEC3) and monocyte-like cells (hNEC4), according to their expression of Colony Stimulating Factor 1 (CSF1), Msh Homeobox 1 (MSX1), Aldo–keto reductase family 1 member C (AKR1C1) (macrophage markers) [113], CXCL1, FERM Domain Containing 4A (FRM4A), C-X-C Motif Chemokine Ligand 6 (CXCL6) (neutrophil markers) [114, 115], and Carboxymethylenebutenolidase homolog (CMBL), Proline Rich and Gla Domain 3 (PRRG3) and Hemicentin 1 (HMCN1) (monocyte markers) [116]. We classified an additional population of myeloid cells as dendritic cells (hNEC6), given the expression of classical markers such as Basic Leucine Zipper ATF-Like Transcription Factor 3 (BATF3), Membrane-spanning 4-domains subfamily A member 6A (MS4A6A), and TYROBP (Fig. 5E and Supplementary Fig. S13C). Interestingly, one cluster uniquely expressed the T-lymphocyte marker CD3E (hNEC2) and other T-cell-like markers, such as CD8 alpha chain (CD8A), Granzyme K (GZMK), Granzyme M (GZMM) and GZMA as well as markers that define NKT-like phenotype such as NKG7, Killer cell lectin-like receptor subfamily B member 1 (KLRB1) and Cluster of Differentiation 96 (CD96). These observations taken together support a CD8 + NKT-like lineage identity for hNEC2. We also identified one cluster of cells expressing MS4A1 and BLNK mRNAs, defined as a cluster of B-cells (hNEC8).

Our analysis also identified non-immune, non-epithelial populations of human breast resident cells, namely Endothelial cells (hNEC7), Fibroblasts (hNEC9) and Adipocytes (hNEC5), which were characterized by markers such as CLDN5, Serum deprivation-response protein (SDPR), and SRY-related HMG-box (SOX18) (endothelial cells) CLD, MYLK, Phospholamban (PLN) and Integrin alpha-7 (ITGA7) (fibroblasts) [22, 103, 117–119], and Phospholipase A2 Group VII (PLA2G7), Leptin Receptor (LEPR), and WNT1-inducible-signaling pathway protein 2 (WISP2) (adipocytes) [120, 121] (Fig. 5E and Supplementary Fig. S13C). These results illustrate the diversity of immune and stromal cells in the normal breast and provides gene signatures that differentiate cells from other lineages, a resource that may enable the identification of cell types and their relevance in normal breast biology.

Collectively, our whole-tissue sequencing approach and re-clustering strategies have improved our understanding of cellular lineages that reside non-cancerous, nulliparous, human breast tissue.

## The Evolutionary Conserved Basis of Murine and Human Breast Epithelial Identity

Our scRNA-seq re-clustering strategy and gene signatures allowed for the identification of diversity across resident cells from the murine and human breast. More specifically, our analysis indicated similar mammary epithelial cellular hierarchy and lineage commitment across species, supporting a body of research that has long utilized mouse models to understand basic process of normal and malignant development. Therefore, we next utilized scRNA-seq profiles from murine and nulliparous human breast epithelial tissue to define the relationships across distinct cell populations and identities across species. Clustering analysis resolved fifteen clusters, represented with cells obtained from murine scRNA-seq datasets (cells yielded from Protocol #1 and Protocol #2) and datasets generated from human breast tissue obtained from healthy, nulliparous, women (five hTM datasets, NgNC Ind #5, NgNC Ind #6, and NgNC Ind #7 datasets, [20] (Supplementary Fig. S14A, B, C, and D and Supplementary File 10).

**Fig. 6:**
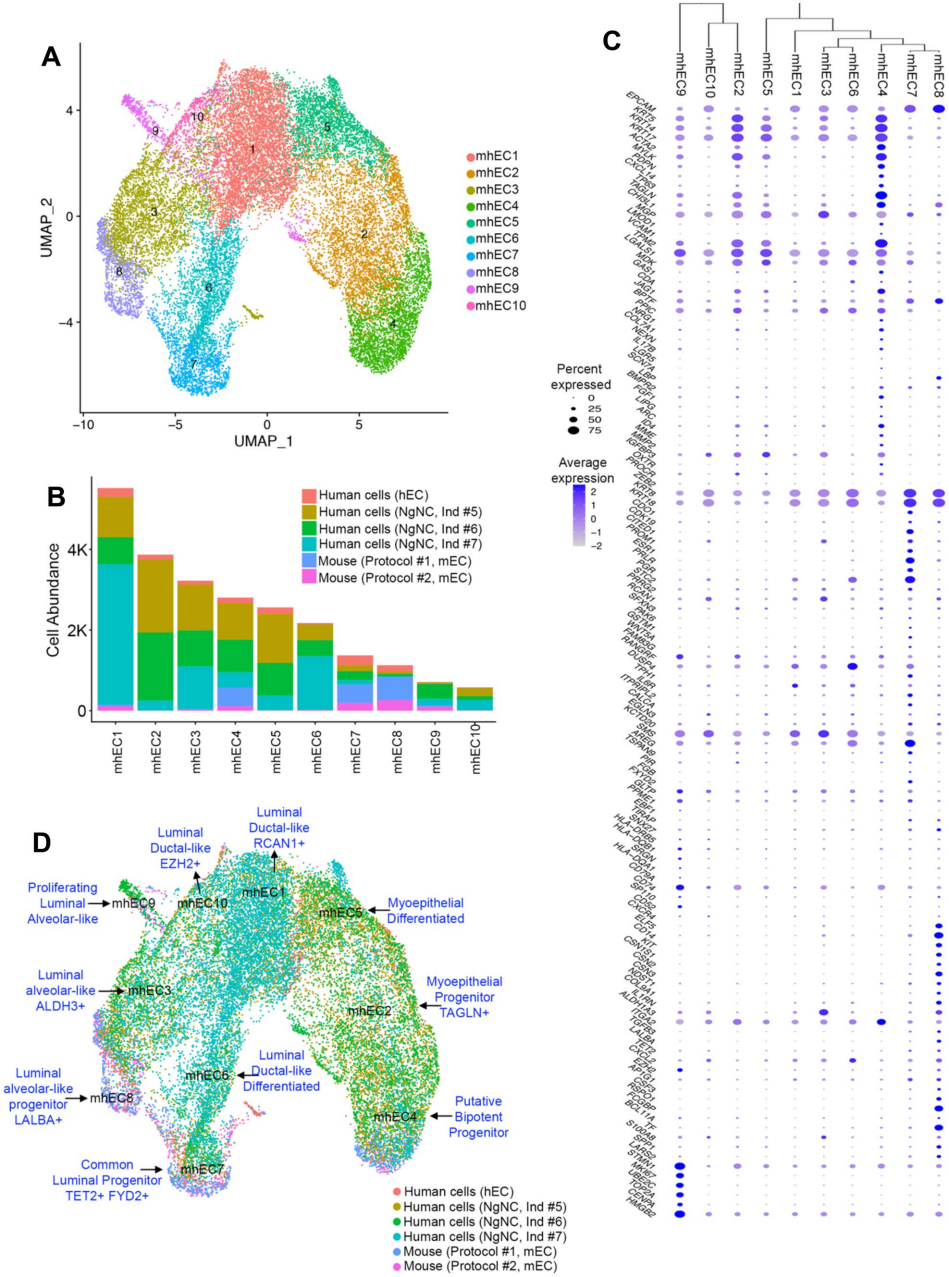
**The evolutionary conserved basis of murine and human breast epithelial identity. (A)** UMAP plot showing the distribution of epithelial cells identified to be present in murine and human breast tissue datasets (mhEC). (**B**) Cell abundance distribution of murine (Protocol #1 and Protocol #2, mEC) and human (hEC, NgNC Ind #5, NgNC Ind #6, NgNC Ind #7) breast tissue datasets. (**C**) Dot plot and dendrogram branching showing the average and percentage of expressed genes that distinguished and classified clusters of epithelial cell lineages present in murine and human breast tissue datasets. (**D**) UMAP plot illustrating the lineage identity of epithelial cell lineages present in murine and human breast tissue datasets

Clustering of merged human and murine cells expressing the epithelial markers EPCAM, KRT8, KRT18, KRT5 and KRT14 (mhTC2, mhTC4, mhTC5, mhTC6, mhTC7, mhTC8, mhTC10, mhTC11, mhTC12), yielded ten clusters of epithelial cells (Fig. 6A and Supplementary Fig. S15A, B). Cell abundancy analysis indicated that all datasets were to some extent represented across all ten mhEC clusters (Fig. 6B and Supplementary Fig. S15C). Clusters mhEC1, mhEC2, mhEC3, mhEC5, mhEC6 and mhEC10 bared higher human-MEC cell abundance, and gene signatures that support their classification as myoepithelial lineages (mhEC2 and mhEC5), and luminal ductal-like RCAN + MECs (mhEC1, mhEC6 and mhEC10) (Fig. 6C, D). Cluster mhEC3 was identified as luminal ductal-like RCAN + KRT14 + MECs, a population that may represent a previously described subset of lobular luminal cells [122], alluding to the usefulness of scRNA-seq analysis into defining breast epithelial cells with specific spatial distribution breast tissue. Moreover, we also identified cluster mhEC8 as being biased towards a murine MEC-fate, a population of luminal-like cells with higher expression of LALBA mRNA (Fig. 6B, C, Supplementary Fig. S3A and Supplementary Fig. S4D).

Clusters evenly composed of murine and human cells spanned several MEC progenitor-like, lineage identities (Fig. 6C, D). Molecular signatures classified cells under cluster mhEC4 as potential bipotent MECs, given the expression of genes that mark both myoepithelial and luminal lineage identities. A population of epithelial cells (cluster mhEC7) was marked by higher levels of TET2 mRNA abundant levels and expression of previously described genes that support both ductal-like hormone sensing and alveolar-like secretory identities, suggesting a luminal common progenitor identity (Fig. 6C, D).

Collectively, our comparative analysis of breast epithelial cells from mouse and human tissue supports the relevance of an expanded gene signature to define lineage identity and represents an initial attempt into understanding the evolutionary conservation of breast epithelial heterogeneity across mice and humans, and their specific relevance during breast development.

## Discussion

Differential tissue fractionation techniques, along with gene signatures published from studies that investigate tissue cellular heterogeneity have independently proven to be valuable tools in defining minute differences between cell populations and lineages. In this study, we describe a strategy that exploits the benefits of both these methodologies, thus avoiding cell-specific isolation, and allowing a deeper understanding of the differences and similarities that define lineage hierarchies and cellular heterogeneity in complex, dynamic tissues such as the mammary gland.

Using this approach, we expanded gene signatures that defined an array of epithelial and non-epithelial cell populations from murine and human mammary gland tissue. The characterization of such gene signatures identified the presence of small populations of mammary bipotential, common progenitor cells, possibly at the apex of the hierarchical tree, and populations of lineage-specific progenitors and subsequent, differentiated epithelial cells. Moreover, these signatures also allowed for the identification of cell populations in cases were previously defined lineage markers were expressed at low levels. For example, our expanded gene signature enabled the identification of bipotential-like MECs, independently of the low levels of *Procr* mRNA, a marker of stem-like mammary cells [8]. This example supports the rationale for an expanded gene expression signature, to broadly define cellular states, especially in cases where cell preparation, isolation, or even mouse strain could represent confounding technical variables that may influence the expression of genes and the classification of MECs.

It is important to note that re-clustering approaches based on cellular transcriptional output may exaggerate population differences without substantial phenotypic relevance. Therefore, to exclude such biases in our analyses, we validated our analytical approach and gene expression signatures in multiple datasets that profiled murine and human mammary epithelial cells. Such comparative analysis confirmed that our analytical approach and molecular signatures enable the identification of MEC identities across all analyzed datasets.

Our analyses may also be useful during the development of transgenic systems to define essential drivers of cell survival, signaling and cellular identity/lineage during mammary gland development. Our expanded expression signatures identified lineage-biased genes that can be used as drivers of lineage tracing strategies and single molecule mathematical predictions. These strategies, in conjunction with single-cell transcriptomics, are instrumental in resolving long-standing questions regarding mammary epithelial cell hierarchy and stemness.

Further highlighting the importance of our strategy, we were able to identify a series of immune and stromal cell populations that reside in murine and human mammary tissue, including rare cell populations, like NKT-like cells and adipocytes. Such analyses allowed for the elaboration of gene signatures that may be utilized in additional scRNA-seq profiling studies to provide a glimpse of their functional characteristics across different stages of mammary gland development. In addition, our ability to profile and identify epithelial and non-epithelial cells in mammary tissue from never pregnant female mice allowed for the analysis of gene signatures that predict parity state [123–126]. Our findings elucidated that such signatures were not restricted to epithelial cells alone, but also extended to non-epithelial cell populations, raising the hypothesis that pregnancy signals change the transcriptional output, and perhaps function, of all mammary resident cells.

Our study of mammary epithelial and non-epithelial cells was also extended to understand the cellular heterogeneity of human breast tissue. We found substantial concurrence in the gene signatures we defined, for epithelial and non-epithelial cell lineages, across both species, supporting the functional conservation of molecular processes across mammalian evolution. Further experimental validation of these defined signatures, using organoid cultures or humanized mammary transplant models, will be invaluable in advancing our understanding of the functional relevance of cellular lineage hierarchies and transitional dynamics, in mouse and human breast tissue. Most importantly, exploitation of these strategies will enable the understanding of cellular dynamics and transcriptional alterations brought to MECs by pregnancy hormones, thus providing comparative, lineage-defining approaches to understand mammary development across mammalian species.

Finally, the utilization of comprehensive gene signatures, comparative analysis not only allow for epithelial cell type identification that is conserved in murine and human breast tissue, but also revealed cell populations that are exclusive to each mammalian system. Interestingly, our results pointed to a more even distribution of stem-like and early progenitor MECs across mouse and human mammary tissue, suggesting the retention of hierarchical points of epithelial cell origin across species, while its evolutionary diversity is represented by more specialized cell types. A deeper dive into how tissue homeostasis, lineage commitment and cellular differentiation is controlled across evolutionary distant mammalian species will improve the interpretation and definition of models that better depict mammary gland function in more evolved species.

By defining strategies that identify commonalities across mouse and breast tissue, our study provides tools and reference signatures that define diversity across differentiation timelines, to enable deeper investigation into the transitional dynamics of normal and malignant mammary gland development.

## Methods

### **Murine Mammary Tissue Processing**

Balb/c female mice (12 – 20 weeks old) were utilized for the generation of scRNAseq profiles. In short, mammary glands (four to five pairs per mouse) were harvested from never pregnant female mice and processed for the selective enrichment of Epithelial cells (Protocol #1 (two mice) = three minutes of mechanic mincing, with 2.5 h enzymatic digestion) or for enrichment of Non-Epithelial cells (Protocol #2 (two mice) = one minute of mechanic mincing, with one-hour enzymatic digestion) with 1 × Collagenase/Hyaluronidase (10 × solution, Stem Cell Technology) at 37o.C (constant agitation) in RPMI 1640 GlutaMAX supplemented with 5% FBS. Digested mammary tissue was washed with cold HBSS supplemented with 5% FBS, followed by incubation with TrypLE Express (Thermo Fisher, #12,604–013) and an additional HBSS wash. Cells were then incubated with Dispase (Stem Cell Technology) supplemented with 40U DNAse I (Sigma, #D4263) for two minutes and filtered through a 100∝m Cell Strainer (BD Falcon, #352,360). All animals were housed at a 12 light/12 dark cycle, with a controlled temperature of 72 °F and 40- 60% of humidity. All experiments were performed in agreement with approved CSHL Institutional Animal Care and Use Committee (IACUC).

### **Antibodies**

The following antibodies were used for the flow cytometric validation of DEG markers. All antibodies were used without further purification. Antibodies for lineage depletion: biotinylated anti-CD45 (eBioscience, #13–0451-85, 1:100 dilution), biotinylated anti-CD31 (eBioscience, #13–0311-85, 1:100 dilution), and biotinylated anti-Ter119 (eBioscience, #13–5921- 85, 1:100 dilution). Antibodies for flow cytometry: eFluor450-conjugated anti-CD24 (eBioscience, #48–0242-82, 1:100 dilution), PE-Cy7-conjugated anti-CD29 (eBioscience, #25–0291-82, 1:100 dilution), Alexa Fluor® 647-conjugated anti-Cytokeratin 5 [EP1601Y](Abcam, #ab193895, 1:200 dilution), Alexa Fluor® 594-conjugated anti-Cytokeratin 8 (Santa Cruz Biotechnology, #sc-8020 AF594, 1:20 dilution), APC-conjugated anti-CD133 (BioLegend, #141,208, Dilution 1:40), BV711- conjugated anti-CD61 (BD Biosciences, #740,677, Dilution 1:40), FITC-conjugated anti-CD52 (Santa Cruz Biotechnology, #sc-51560 FITC, Dilution 1:40), PE-conjugated anti-CD79a (Abcam, #ab177274, Dilution 1:40), PE-conjugated anti-p63 (Santa Cruz Biotechnology, #sc-25268 PE, Dilution 1:20), Alexa Fluor® 700-conjugated anti-Lgr5 (R&D Systems, #FAB82401N, Dilution 1:40), Alexa Fluor® 488-conjugated anti-CA II (Santa Cruz Biotechnology, #sc-48351 AF488, Dilution 1:20), APC-conjugated anti-Lalba (LS Bio, #LS-C716395-200, Dilution 1:40), and PE- conjugated anti-Tet2 (Cell Signaling Technology, #79,468, Dilution 1:50). OneComp eBeads™ Compensation beads (Invitrogen, #01–1111-42) were used for negative and positive compensation controls.

### **Flow Cytometry**

 Mammary glands (four per mouse) were harvested, minced and incubated for ~ 2.5 h with 10 × Collagenase/Hyaluronidase in DMEM (Stem Cell Technology, #07,912, Dilution 1:10) in RPMI1640 GlutaMAX™ supplemented with 5% FBS. Digested mammary gland fragments were washed with cold HBSS supplemented with 5% FBS, followed by incubation with pre-warmed (37 ºC), TrypLE Express (Thermo Fisher, #12,604–013) for five minutes at room temperature and an additional HBSS wash. Cells were incubated with 1 mL of Dispase in HBSS (Stem Cell Technology, #07,913, 5 U/ml) supplemented with 40 µL DNAse I (Sigma, #D4263) for two minutes and then filtered through a 100µm Cell Strainer (BD Falcon, #352,360). The single cell suspension was incubated with lineage depletion antibodies and Anti-Biotin MicroBeads (Miltenyi Biotec, #130–090-485), followed by loading onto MACS LS column (Miltenyi Biotec, #130–042-401). Flow- through cells (lineage negative, epithelial cells) were collected and stained with antibodies against surface antigens for 40 min at 4 ºC. The stained cells were washed in 1X MACs buffer and fixed in 1% PFA for 20 min at room temperature. Cells were then permeabilized using Invitrogen eBioscience™ Foxp3/Transcription Factor Staining Buffers (Invitrogen #00–5523-00) and stained with antibodies against intracellular antigens diluted in Invitrogen 1 × Perm/wash buffer for 40 min at room temperature. Surface and intracellular stained cells were re-suspended in 1X MACs buffer and filtered prior to acquisition. Flow cytometry acquisition was carried out on the Dual Fortessa II cell analyzer (BD Bioscience). Data analysis was performed using FACSDiva™ 8 software (BD) and FlowJo™ Software (BD).

### **Human Mammary Tissue Processing**

Non-identified, non-cancerous, human breast tissue (n = 5) was obtained from healthy, nulliparous women undergoing cosmetic breast reduction surgery via the Northwell Health Tissue Donation Program (TDP). Surgically removed tissue was minced for five minutes and digested with 1 × Collagenase/Hyaluronidase (10 × solution, Stem Cell Technology) at 37o.C (constant agitation) in RPMI 1640 GlutaMAX supplemented with 5% FBS, for 4–6 h. Digested mammary tissue was washed with cold HBSS supplemented with 5% FBS, followed by incubation with TrypLE Express (Thermo Fisher, #12,604–013) and an additional HBSS wash. Cells were then incubated with Dispase (Stem Cell Technology) supplemented with 80U DNAse I (Sigma, #D4263) for two minutes and filtered through a 100µm Cell Strainer (BD Falcon, #352,360). Tissue collection and handling performed in agreement with approved CSHL Institutional Review Board (IRB).

### **scRNA-Seq Library Preparation**

For the mouse mammary tissue analysis, five thousand total mammary cells with a viability of > 90% were used for cDNA synthesis and library preparation utilizing the 10 × Chromium platform. Single-cell libraries were run using single-end sequencing with indexing on a NextSeq 550 high output platform. Human mammary tissue scRNAseq library preparation and sequencing were performed by the New York Genome Center, utilizing in-house developed protocols and sequencers.

### **scRNA-seq Data Analysis**

Murine (two samples, each sample prepared from mammary glands pulled from two mice) and human (five samples) scRNA-seq data were aligned to mm10 and hg19 genomes respectively, using CellRanger version 3 [127] and downstream data processing was performed using Seurat version 3.2.0 [128]. Murine scRNA-seq samples were merged into a single Seurat object (Sobj) as were human scRNA-seq samples. For batch normalization, anchors were found between the merged datasets using the FindIntegrationAnchors() function and then integrated using the IntegrateData() function [129]. For the murine Sobj, 15,359 total murine cells (mTM) were utilized, with quality control steps were taken at each at each re-clustering phase, resulting in the removal of clusters deemed to be low quality based on an average of cells expressing comparatively low or high features or a high percentage of mitochondrial content. For the human Sobj, cells with fewer than 500 or more than 10,220 features were removed, as were cells with greater than 15% mitochondrial content, resulting in 2,053 total human cells (hTM). Similarly, quality control was employed and checked at each re-clustering approach to remove comparatively low-quality clusters. Doublets were identified and removed in both Sobjs. Processing for both datasets started with a principal component analysis (PCA) using the top 2,000 variable genes to identify the number of significant components before clustering. Uniform manifold approximation and projection (UMAP) clustering was performed by calculating a shared nearest neighbor graph (SNN), using a resolution of 0.5. Epithelial cells for both datasets were defined by the expression of Epcam, Krt8, Krt18, Krt5 and Krt14(cluster average expression > 2). Non-epithelial were cells considered having low expression of Epcam, Krt8, Krt18, Krt5 and Krt14. Epithelial and non-epithelial clusters were separated using the subset() function and then formed to new Sobjs after re-clustering. For the murine epithelial and non-epithelial Sobj as well as both human Sobj, re-clustering was performed by calculating a SNN using ten dimensions and a resolution of 0.5. Low quality clusters were identified and removed resulting in 2,016 epithelial cells (EC) and 12,646 non-epithelial cells (NEC) in murine Sobjs, and 440 epithelial cells (hEC) and 1,456 non-epithelial cells (hNEC) in human Sobjs. The FindMarkers() function, which uses a Wilcoxon rank-sum test to identify differentially expressed genes, was implemented to determine differentially expressed genes between clusters. Visualization functions such as DotPlot(), FeaturePlot(), VlnPlot(), and HeatMap() were utilized to examine differentially expressed genes and markers of interest. The BuildClusterTree() function was employed with default parameters to generate dendrograms of clusters. Construction of cellular trajectories within epithelial clusters was conducted using Monocle 3 [130]. The SingleR package was used for annotation of our human Sobj against publicly available datasets which included DICE, HPCA, Monaco and NoverHem, all of which were attained through Bioconductor [93, 131–134]. Retrieval of Bach et al. dataset was achieved through the use of BachMammaryData(), which was then processed into separate Sobjs based on mammary gland developmental timepoints. Following the same procedure previously described, data was clustered and visualized in UMAP plots. Cell identities in the Bach et al. datasets were predicted through use of our generated cell identities in our murine epithelial dataset as a reference when implementing the FindTransferAnchors() function. These anchors were then inputted into the TransferData() function to determine a predicted murine epithelial cluster identification for each cell in the Bach et al. dataset [8, 135]. For cross-species joint scRNA-seq analysis, mouse and human one-to-one orthologs were retrieved from [136]. Mouse gene names were first converted to its orthologous human gene names, then human and mouse samples were merged using Seurat. Cells with fewer than 200 or more than 6000 features were removed for quality control. The final integration of human and mouse samples resulted in 15,200 murine cells and 23,608 human cells as well as 14,928 genes for further analysis. Other parameters used in the processing steps are kept the same as those in murine analysis, including both PCA and UMAP for generating clusters for all cells and re-clustering for epithelial cells. After sub-setting epithelial cells (i.e., cells with high expressions of Epcam, Krt8, Krt18 and Krt5), Monocle 3 was used to construct cellular trajectories within the epithelial clusters. Pathway analysis was performed using Enrichr [137, 138].

## Supplementary Information

Below is the link to the electronic supplementary material.

Supplementary file1 (XLSX 13 KB)

Supplementary file2 (XLSX 421 KB)

Supplementary file3 (XLSX 26 KB)

Supplementary file4 (XLSX 24 KB)

Supplementary file5 (XLSX 441 KB)

Supplementary file6 (XLSX 372 KB)

Supplementary file7 (XLSX 223 KB)

Supplementary file8 (XLSX 19 KB)

Supplementary file9 (XLSX 314 KB)

Supplementary file10 (XLSX 102 KB)

Supplementary file11 (PDF 12119 KB)

## Data Availability

scRNA-seq datasets were deposited into NCBI database [https://www.ncbi.nlm.nih.gov/], Sequence Read Archive SUB8429356, and will be made available upon manuscript acceptance/publication. Additional filtered feature matrix files may be found at https://github.com/dosSantosLabCSHL/JOMG-Henry_Trousdell_Cyrill_Et_al. Previously published datasets for murine mammary epithelial cells are available under the following IDs: GSM2834498, GSM2834499 (mammary tissue from nulliparous C57BL/6 female mice), GSM2834500, GSM2834501 (mammary tissue from C57BL/6 female mice at mid-gestation), GSM2834502, GSM2834503 (mammary tissue from C57BL/6 female mice during lactation), GSM2834504, GSM2834505 (mammary tissue from nulliparous C57BL/6 female mice at late involution stage). Previously published datasets for human breast epithelial cells are available under the following IDs: GSM3099846 (Ind#4 MECs), GSM3099847 (Ind#5 MECs), GSM3099848 (Ind #6 MECs), and GSM3099849 (Ind #7 MECs).
