# Supplementary material for: Characterization of Gene Expression Signatures for the Identification of Cellular Heterogeneity in the Developing Mammary Gland
Source: J Mammary Gland Biol Neoplasia. 2021 May 14;26(1):43–66. doi: 10.1007/s10911-021-09486-3 (PMC8217035; doi:10.1007/s10911-021-09486-3)
Supplement: Supplementary file 11 — Supplementary file11 (PDF 12119 KB) [file 10911_2021_9486_MOESM12_ESM.pdf]

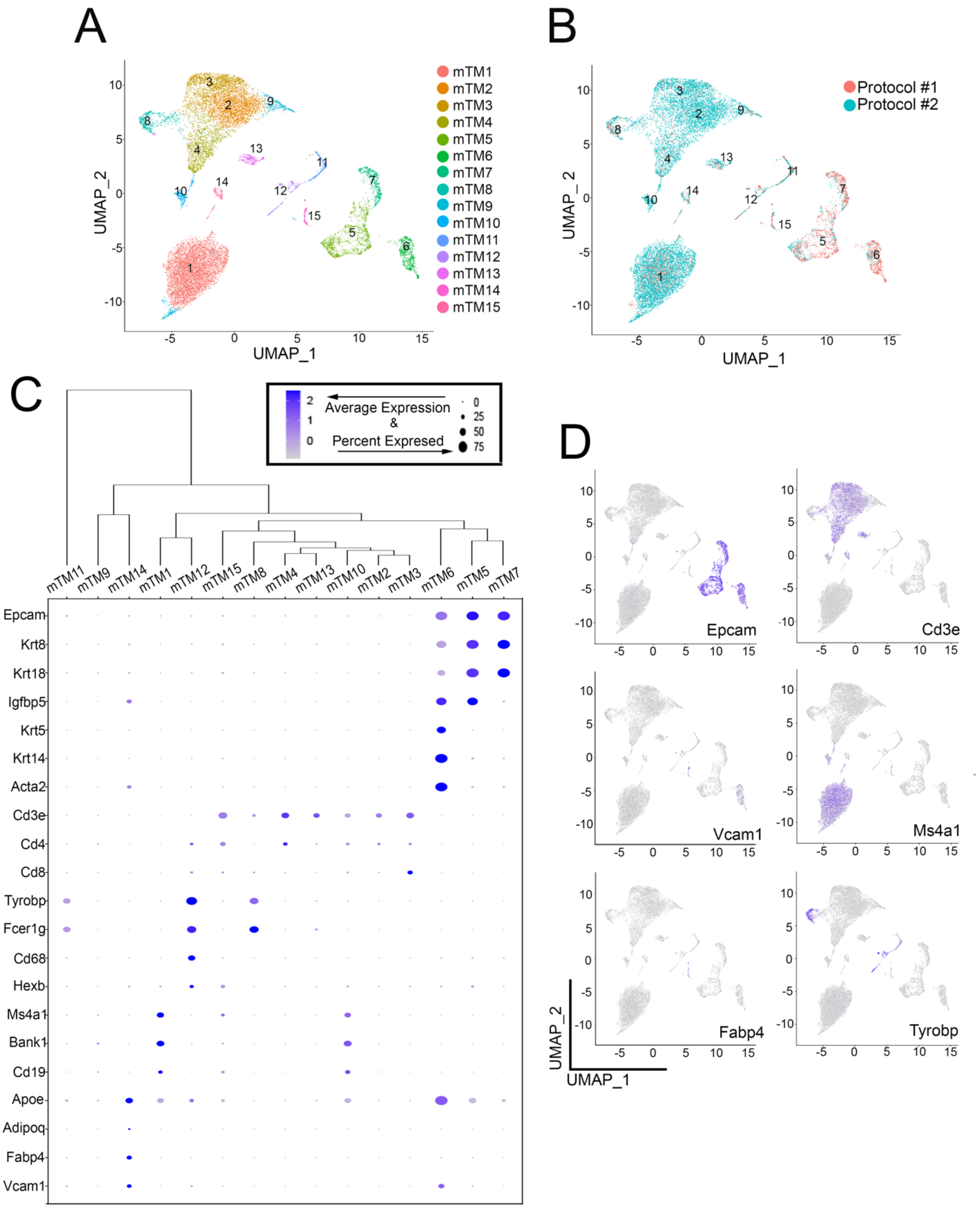
A
B
C
D
UMAP_1
UMAP_2
mTM1
mTM2
mTM3
mTM4
mTM5
mTM6
mTM7
mTM8
mTM9
mTM10
mTM11
mTM12
mTM13
mTM14
mTM15
Protocol #1
Protocol #2
Average Expression
&
Percent Expresed
mTM11 mTM9 mTM14 mTM1 mTM12 mTM15 mTM8 mTM4 mTM13 mTM10 mTM2 mTM3 mTM6 mTM5 mTM7
Epcam
Krt8
Krt18
Igfbp5
Krt5
Krt14
Acta2
Cd3e
Cd4
Cd8
Tyrobp
Fcer1g
Cd68
Hexb
Ms4a1
Bank1
Cd19
Apoe
Adipoq
Fabp4
Vcam1
Epcam
Cd3e
Vcam1
Ms4a1
Fabp4
Tyrobp

Figure 1

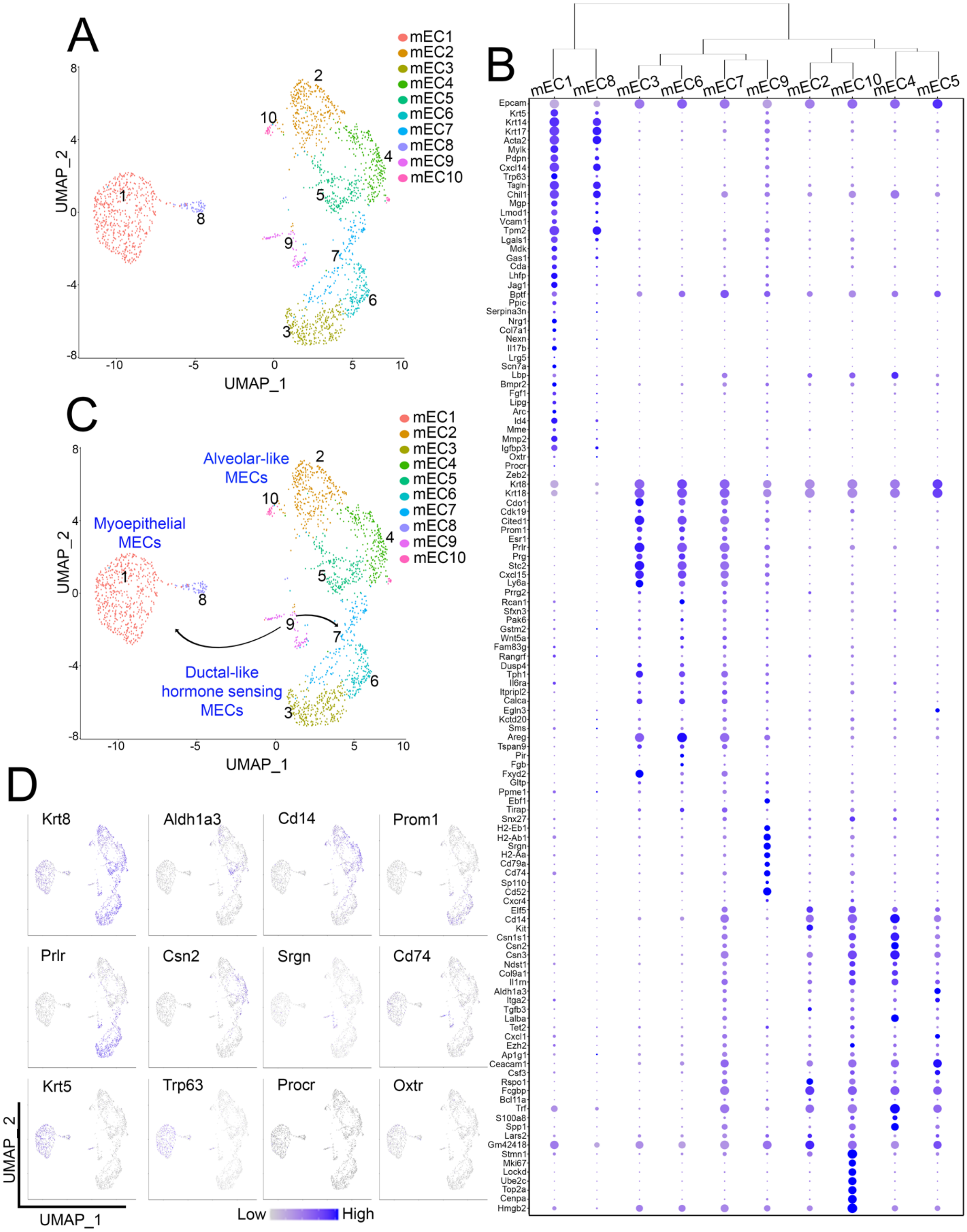
A
B
C
D
mEC1
mEC2
mEC3
mEC4
mEC5
mEC6
mEC7
mEC8
mEC9
mEC10
UMAP_1
UMAP_2
Alveolar-like MECs
Myoepithelial MECs
Ductal-like hormone sensing MECs
Krt8
Aldh1a3
Cd14
Prom1
Prlr
Csn2
Srgn
Cd74
Krt5
Trp63
Procr
Oxtr
Low
High

Figure 2

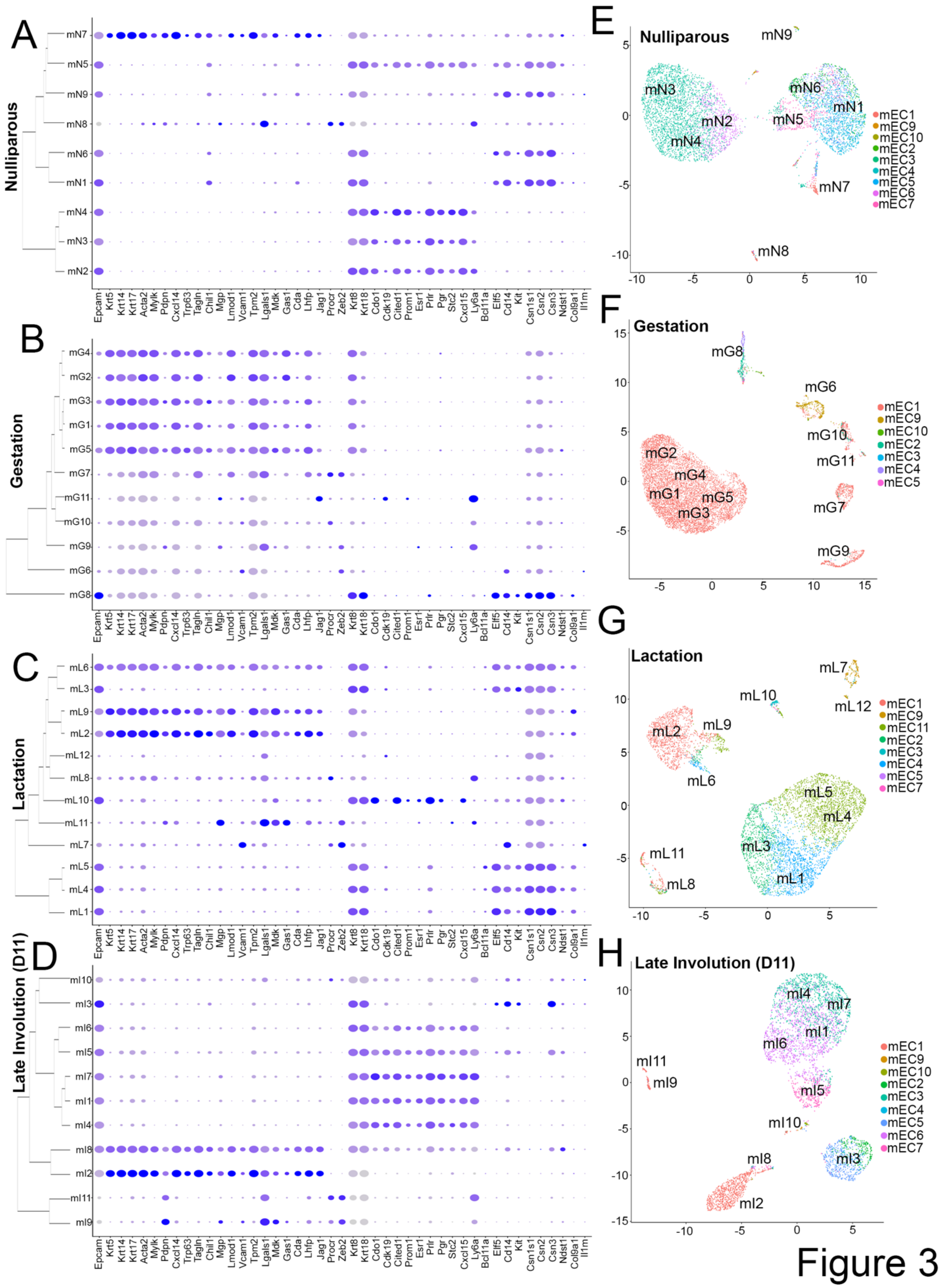
A
B
C
D
E
F
G
H
Nulliparous
Gestation
Lactation
Late Involution (D11)
Epcam Krt5 Krt14 Krt17 Acta2 Mylk Pdpn Cxcl14 Trp63 Tagln Chil1 Mgp Lmod1 Vcam1 Tpm2 Lgals1 Mdk Gas1 Cda Lhfp Jag1 Procr Zeb2 Krt8 Krt18 Cdo1 Cdk19 Cited1 Prom1 Esr1 Prlr Pgr Stc2 Cxcl15 Ly6a Bcl11a Elf5 Cd14 Kit Csn1s1 Csn2 Csn3 Ndst1 Col9a1 Il1m
mEC1 mEC9 mEC10 mEC2 mEC3 mEC4 mEC5 mEC6 mEC7
mEC11

Figure 3

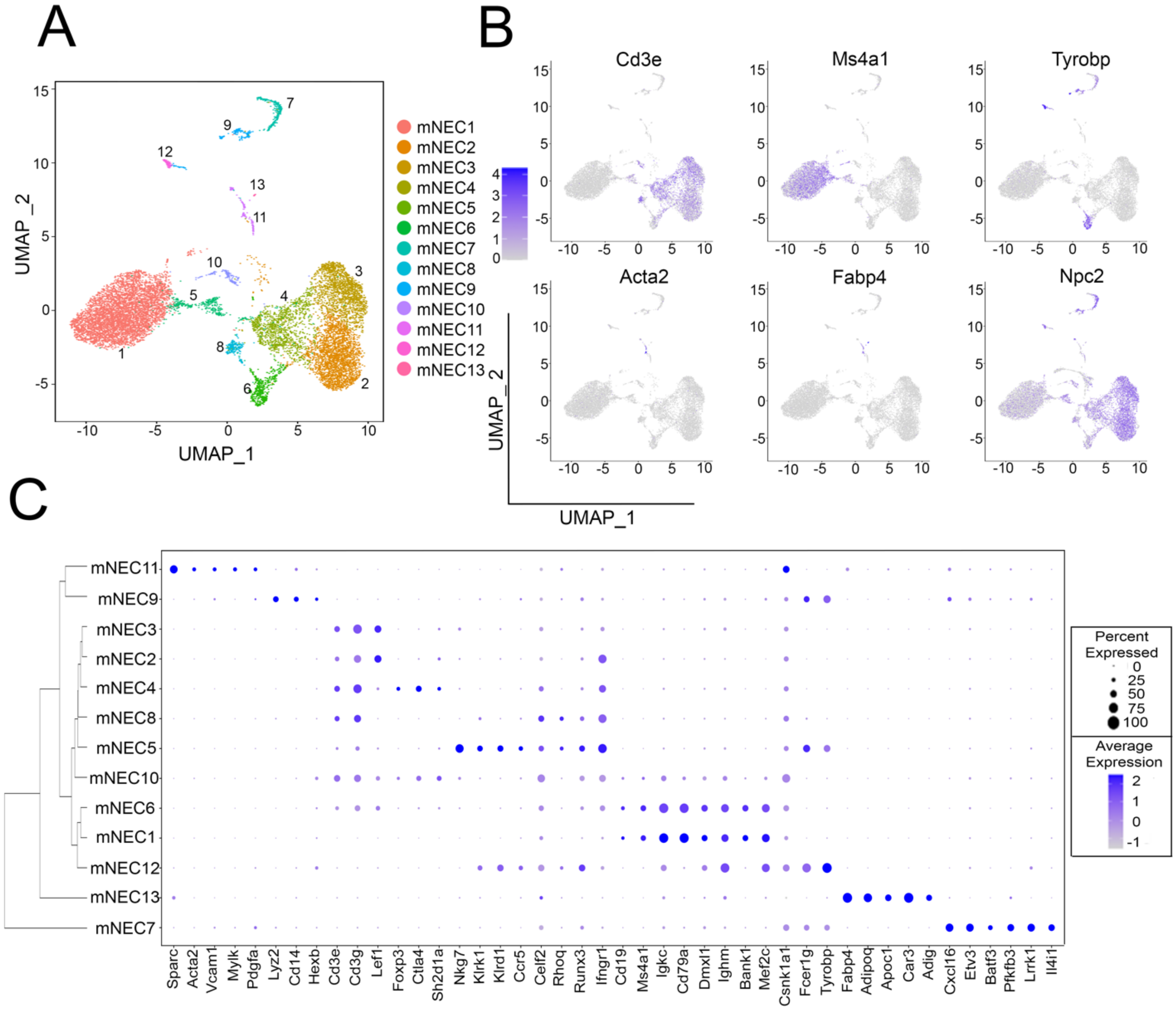
A
B
C
UMAP_1
UMAP_2
mNEC1
mNEC2
mNEC3
mNEC4
mNEC5
mNEC6
mNEC7
mNEC8
mNEC9
mNEC10
mNEC11
mNEC12
mNEC13
Cd3e
Ms4a1
Tyrobp
Acta2
Fabp4
Npc2
Percent Expressed
0
25
50
75
100
Average Expression
2
1
0
-1
Sparc
Acta2
Vcam1
Mylk
Pdgfa
Lyz2
Cd14
Hexb
Cd3e
Cd3g
Lef1
Foxp3
Ctla4
Sh2d1a
Nkg7
Klrk1
Klrd1
Ccr5
Celf2
Rhoq
Runx3
Ifngr1
Cd19
Ms4a1
Igkc
Cd79a
Dmxl1
Ighm
Bank1
Mef2c
Csnk1a1
Fcer1g
Tyrobp
Fabp4
Adipoq
Apoc1
Car3
Adig
Cxcl16
Etv3
Batf3
Pfkfb3
Lrrk1
Il4i1

Figure 4

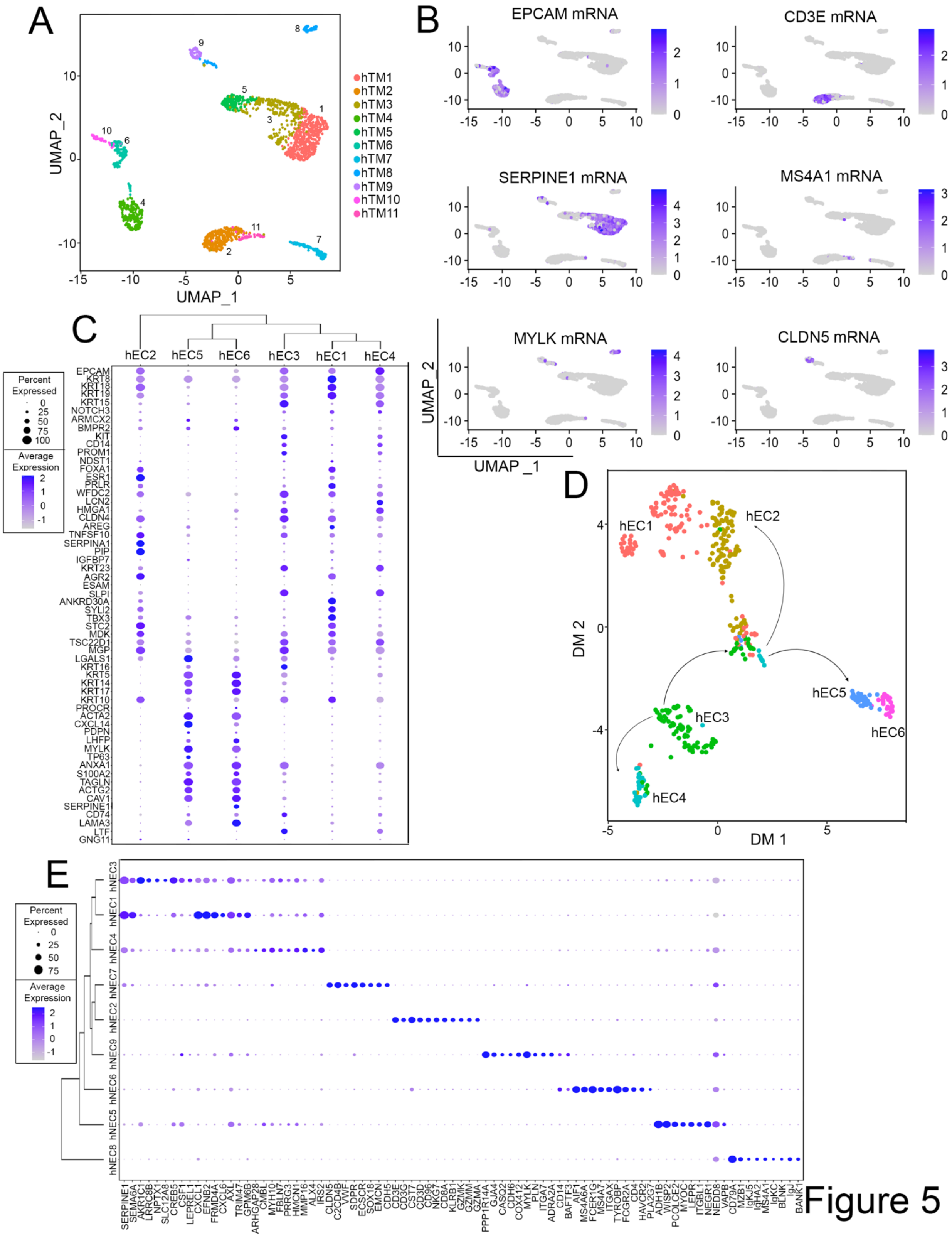
A
UMAP_1
UMAP_2
hTM1
hTM2
hTM3
hTM4
hTM5
hTM6
hTM7
hTM8
hTM9
hTM10
hTM11
B
EPCAM mRNA
CD3E mRNA
SERPINE1 mRNA
MS4A1 mRNA
MYLK mRNA
CLDN5 mRNA
UMAP_2
UMAP _1
C
hEC2
hEC5
hEC6
hEC3
hEC1
hEC4
Percent Expressed
Average Expression
EPCAM
KRT8
KRT18
KRT19
KRT15
NOTCH3
ARMCX2
BMPR2
KIT
CD14
PROM1
NDST1
FOXA1
ESR1
PRLR
WFDC2
LCN2
HMGA1
CLDN4
AREG
TNFSF10
SERPINA1
PIP
IGFBP7
KRT23
AGR2
ESAM
SLPI
ANKRD30A
SYLI2
TBX3
STC2
MDK
TSC22D1
MGP
LGALS1
KRT16
KRT5
KRT14
KRT17
KRT10
PROCR
ACTA2
CXCL14
PDPN
LHFP
MYLK
TP63
ANXA1
S100A2
TAGLN
ACTG2
CAV1
SERPINE1
CD74
LAMA3
LTF
GNG11
D
DM 1
DM 2
hEC1
hEC2
hEC3
hEC4
hEC5
hEC6
E
Percent Expressed
Average Expression
hNEC3
hNEC1
hNEC4
hNEC7
hNEC2
hNEC9
hNEC6
hNEC5
hNEC8

Figure 5

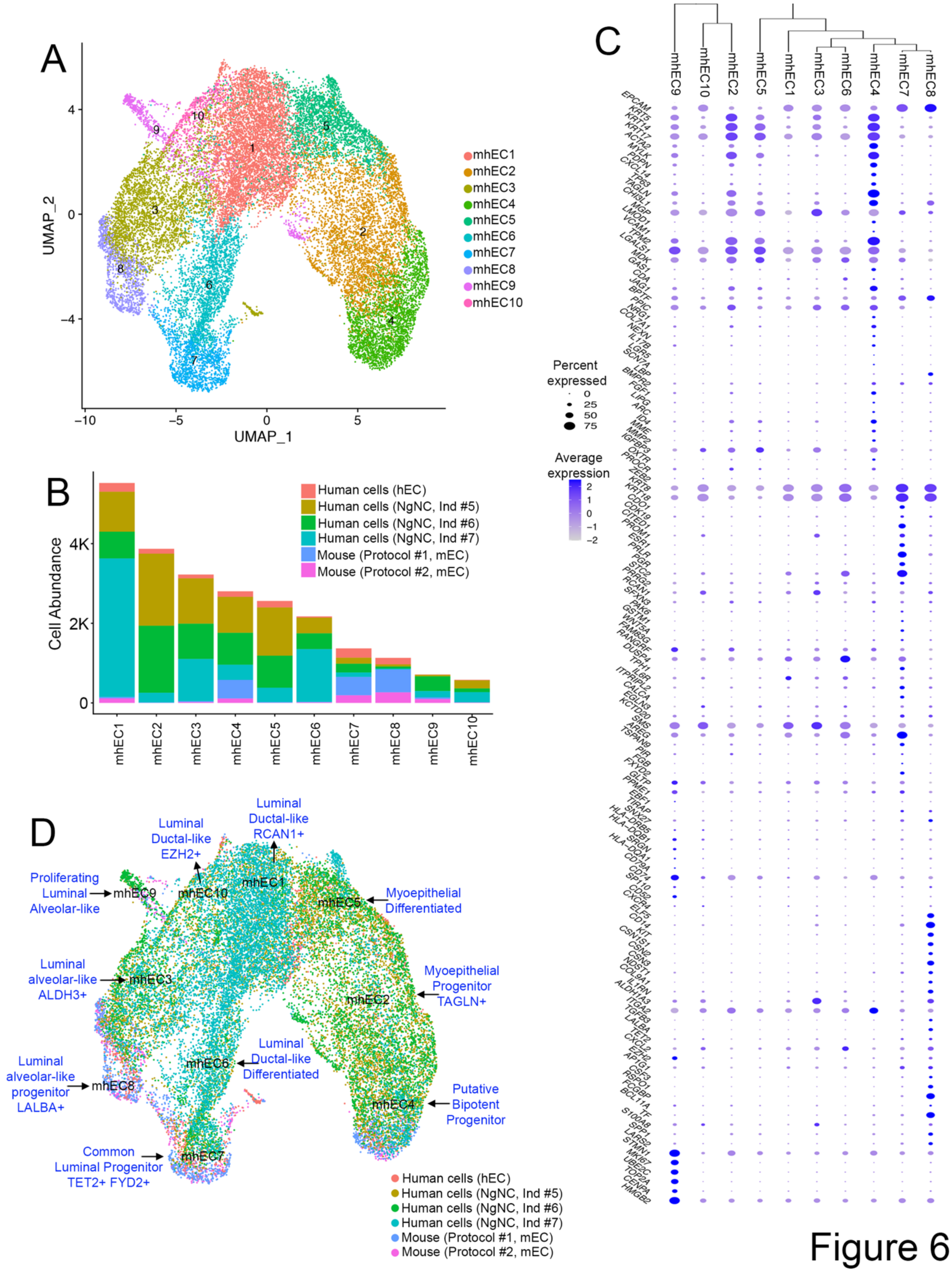
A
UMAP_1
UMAP_2
mhEC1
mhEC2
mhEC3
mhEC4
mhEC5
mhEC6
mhEC7
mhEC8
mhEC9
mhEC10
B
Cell Abundance
Human cells (hEC)
Human cells (NgNC, Ind #5)
Human cells (NgNC, Ind #6)
Human cells (NgNC, Ind #7)
Mouse (Protocol #1, mEC)
Mouse (Protocol #2, mEC)
C
Percent expressed
Average expression
D
Proliferating Luminal Alveolar-like
Luminal Ductal-like EZH2+
Luminal Ductal-like RCAN1+
Myoepithelial Differentiated
Luminal alveolar-like ALDH3+
Myoepithelial Progenitor TAGLN+
Luminal Ductal-like Differentiated
Luminal alveolar-like progenitor LALBA+
Putative Bipotent Progenitor
Common Luminal Progenitor TET2+ FYD2+

Figure 6

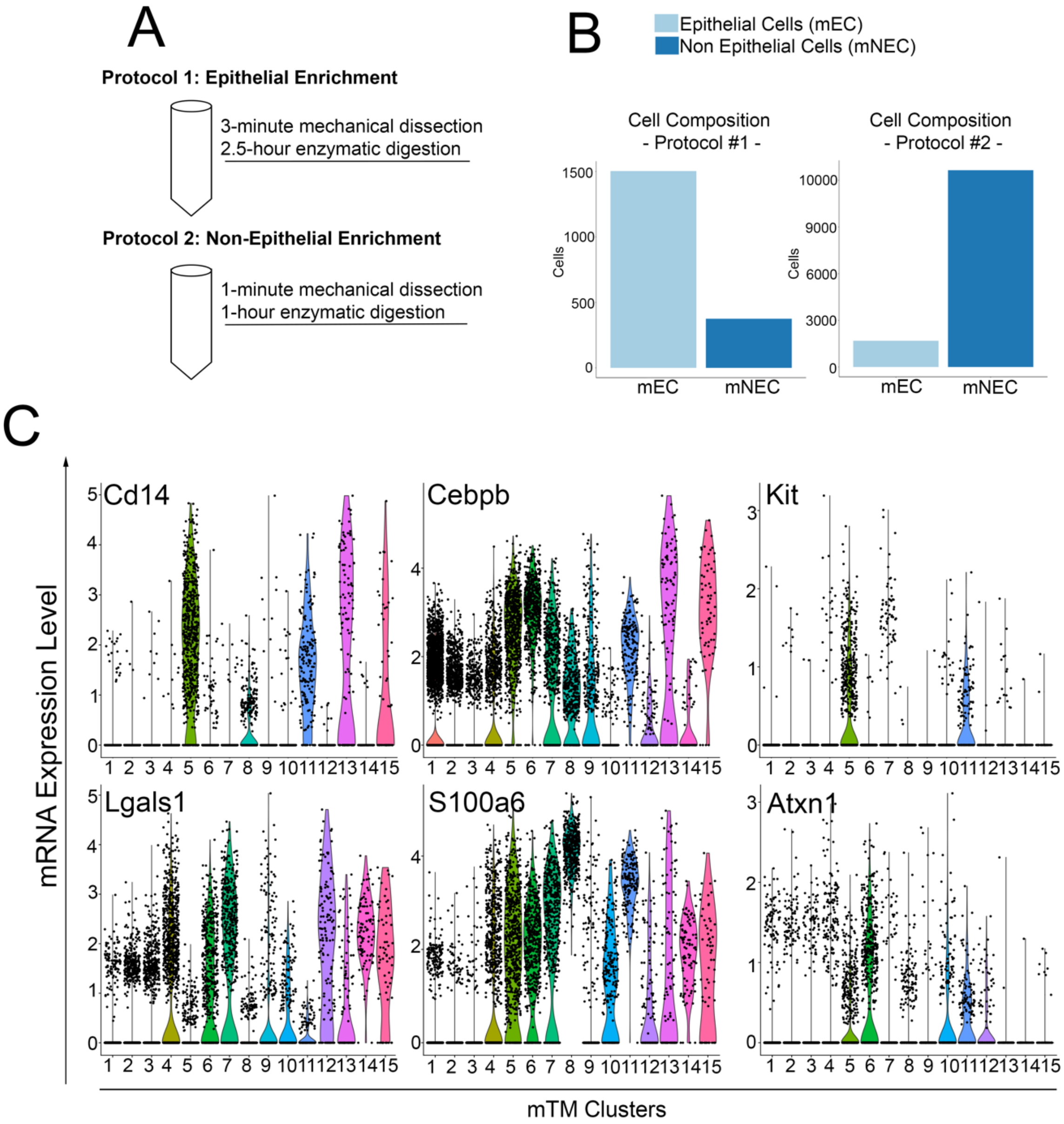

Supplementary Fig. S1

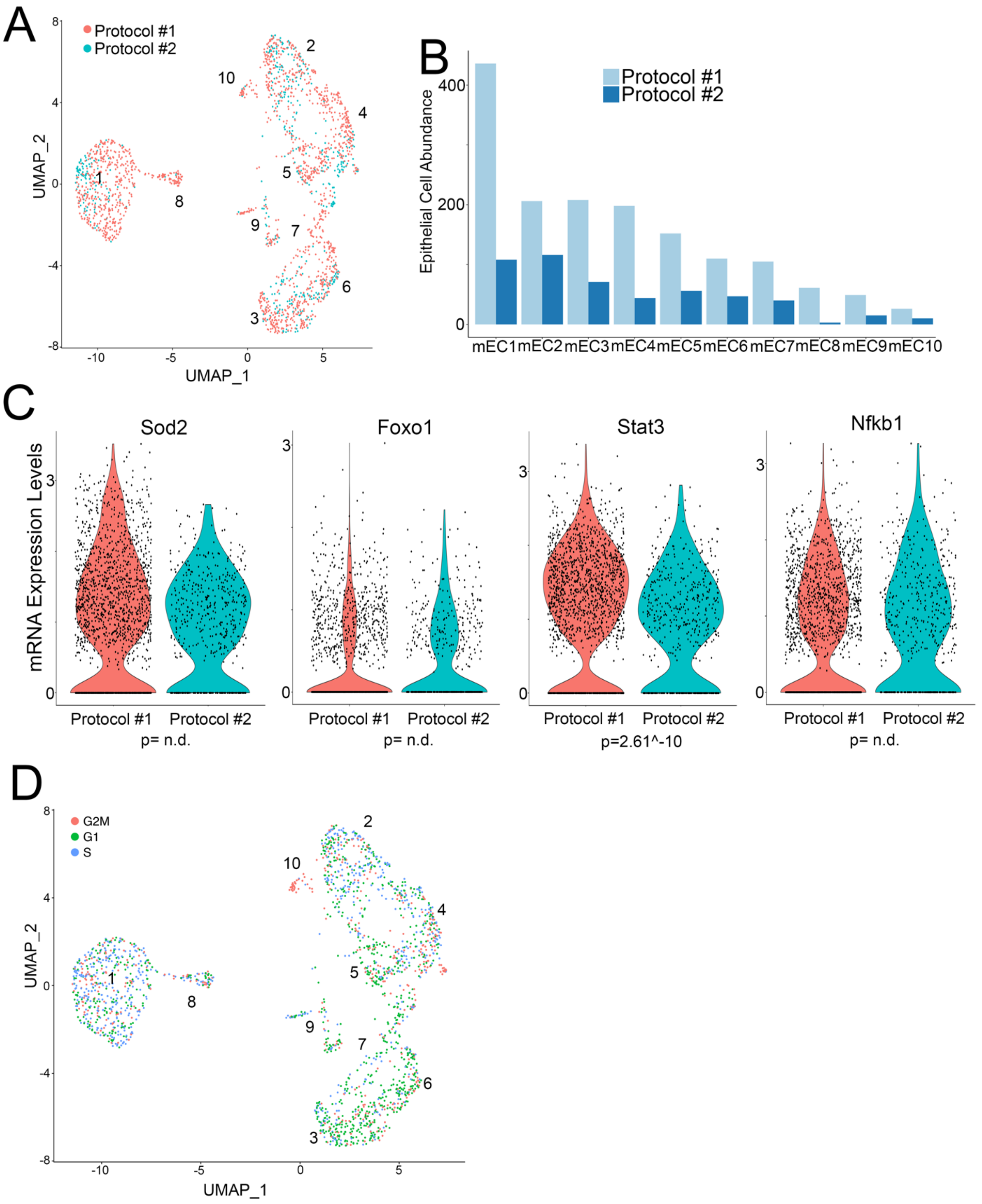

Supplementary Fig. S2

A

Epithelial Cluster	Epithelial Cell Lineage	Lineage-associated genes
mEC1	Myoepithelial Progenitor	Epcam(low), Lgals1, Bptf, Krt17, Ppic, Mdk, Krt14, Krt5, Acta2, Mgp, Lmod, Lhfp, Cxcl14, Serpina3n, Cnn1, Vcam1, Nrg1, Col7a1, Nexn, Il17b, Mylk, Sparc, Lgr5, Jag1, Scn7a, Trp63, Lbp, Tagln, Bmpr2, Fgf1, Lipg, Arc, Id4, Mme, Mmp2, Igfbp3
mEC2	Luminal hormone negative progenitor	Epcam, Krt8, Krt18, Rspo1, Col9a1, Aldh1a3, Il1rn, Csn1s1, Tgfb3, Bptf, Kit, Cxcl1, Ndst1, Cd14, Gjb2, Tagln, Ppme1, Notch3, Parp1, Bcl11a, Fcgbp
mEC3	Luminal ductal-like differentiated Fxyd2+	Epcam, Krt8, Krt18, Prlr, Prrg2, Ak3, Cdk19, Fxyd2, Areg, Stc2, Prom1, Esr1, Pgr, Cdo1, Gstm2, Wnt5, Cxcl15, Stc2,Ly6a, Tspan9, Gltp, Cd14, Ppme1, Adck5, Dusp4, Tph1, Notch3, Itpripl1, Calca
mEC4	Luminal alveolar-like progenitor Lalba+	Epcam, Krt8, Krt18, Col9a1, Il1rn, Itga2, Csn1s1, Car2, Csn2, Bptf, Lalba, Kit, Armcx2, Trf, Cxcl1, Ndst1, Ezh2, Ap1g1, Areg, Spp, Sfxn3, Cd14, Snx27, Mfsd5, S100a8, Lbp, Gjb2, Notch3, Il6ra, Kctd20, Erf, Ptbp2, Ireb2
mEC5	Luminal alveolar-like Aldh1a3+	Epcam, Krt8, Krt18, Col9a1, Aldh1a3, Il1rn, Itga2, Csn1s1, Car2, Csn2, Ceacam1, Bptf, Kit, Armcx2, Csf3, Cxcl1, Ndst1, Ezh2, Ap1g1, Areg, Cd14, Snx27, Lbp, Bmpr2, Egln3, Erf, Ptbp2
mEC6	Luminal ductal-like differentiated Rcan1+	Epcam, Krt8, Krt18, Prlr, Armcx2, Ak3, Cdk19, Cited1, Areg, Stc2, Rcan1, Prom1, Esr1, Pgr, Pak6, Cdo1, Wnt5, Cxcl15, Ly6a, Tspan9, Pir, Fgb, Cd14, Fam83g, Dusp4, Tph1, Notch3, Il6ra, Itpripl2, Calca, Ptbp2
mEC7	Common Luminal progenitor Tet2+	Epcam, Krt8, Krt18, Prlr, Col9a1, Aldh1a3, Il1rn, Itga2, Csn1s1, Car2, Lgals1, Csn2, Tgfb3, Bptf, Lalba, Kit, Armcx2, Tet2, Cxcl1, Ndst1, Ezh2, Ap1g1, Prrg2, Ak3, Cdk19, Cited1, Stc2, Rcan1, Esr1, Pgr, Sfxn3, Pak6, Cdo1, Gstm2, Wnt5, Cxcl15, Ly6a, Cd14, Snx27, Mfsd5, Lbp, Gjb2, Bmpr2, Fam83g, Rangrf, Adck5, Dusp4, Tph1, Notch3, Il6ra, Itpripl2, Calca, Egln3, Kctd20, Erf, Setd7, Cwc22, Ptbp2, Ireb2, Parp1, Sms
mEC8	Myoepithelial differentiated	Epcam(low), Lgals1, Krt17, Krt14, Krt5, Acta2, Mgp, Lmod, OxtR, Cxcl14, Cnn1, Mylk, Sparc, Tagln, Bmpr2, Igfbp3
mEC9	Bipotential Progenitor	Epcam, Krt8, Krt18, Prlr, Lgals1, Bptf, Krt17, Cited1, Acta2, Tir, Pgr, Ebf1, Cdo1, Cxcl15, Ly6a, H2-eb1, H2-ab1, Srgn, H2-Aa, Gltp, Krt14, Krt5, Lhfp, Cxcl14, Mylk, Sparc, Jag1, Tagln, Bmpr2, Ppme1, Dusp4, Tph1, Calca, Sms, CD79a, CD74, Ctss, Sp110, Cd52
mEC10	Proliferating Luminal alveolar-like	Epcam, Krt8, Krt18, Col9a1, Il1rn, Itga2, Ireb2, Csn1s1, Car2, Lgals1, Stmn1, Csn2, Tgfb3, mki67, Lalba, Lockd, Kit, Armcx2, Cxcl1, Ndst1, H2afz, Ezh2, Ap1g1, Ube2c, Prrg2, Ak3, Cdk19, Areg, Sfxn3, Mdk, Ly6a, Krt14, Cd14, Snx27, Mfsd5, Top2, Lbp, Gjb2, Tagln, Cenpa, Bmpr2, Fam83g, Rangrf, Ppme1, Notch3, Hmgb2, Il6ra, Itpripl2, Kctd20, Erf, Setd7, Cwc22, Ptbp2, Ireb2, Parp1, Sms, Sp110, Cxcr4

B

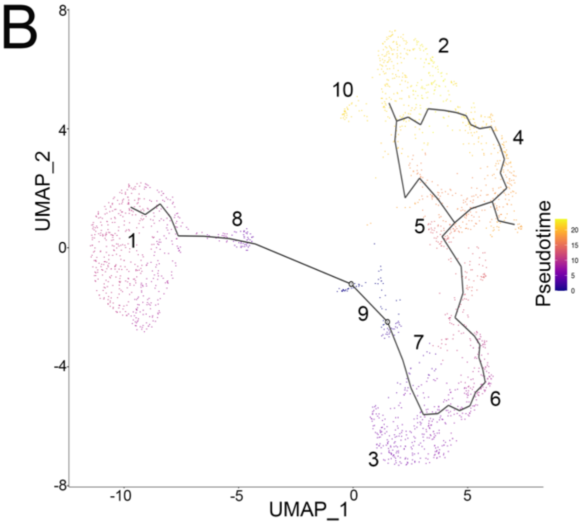

C

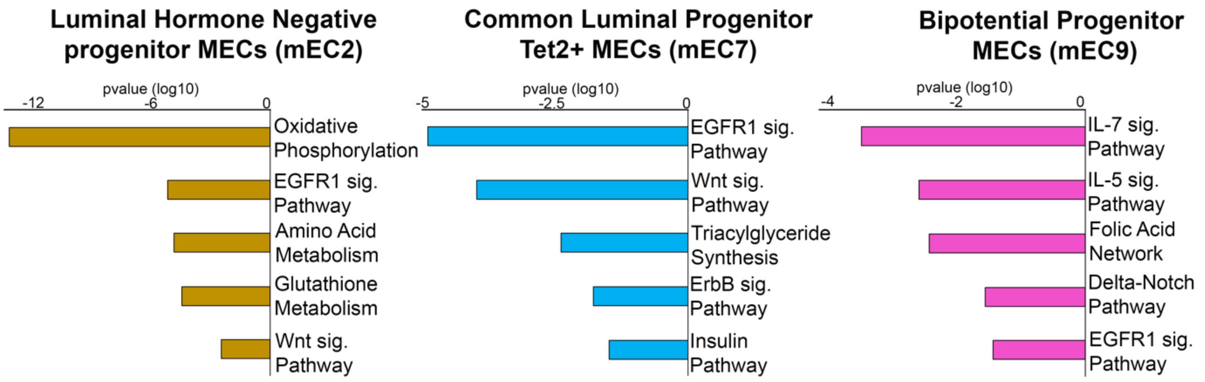

Supplementary Fig. S3

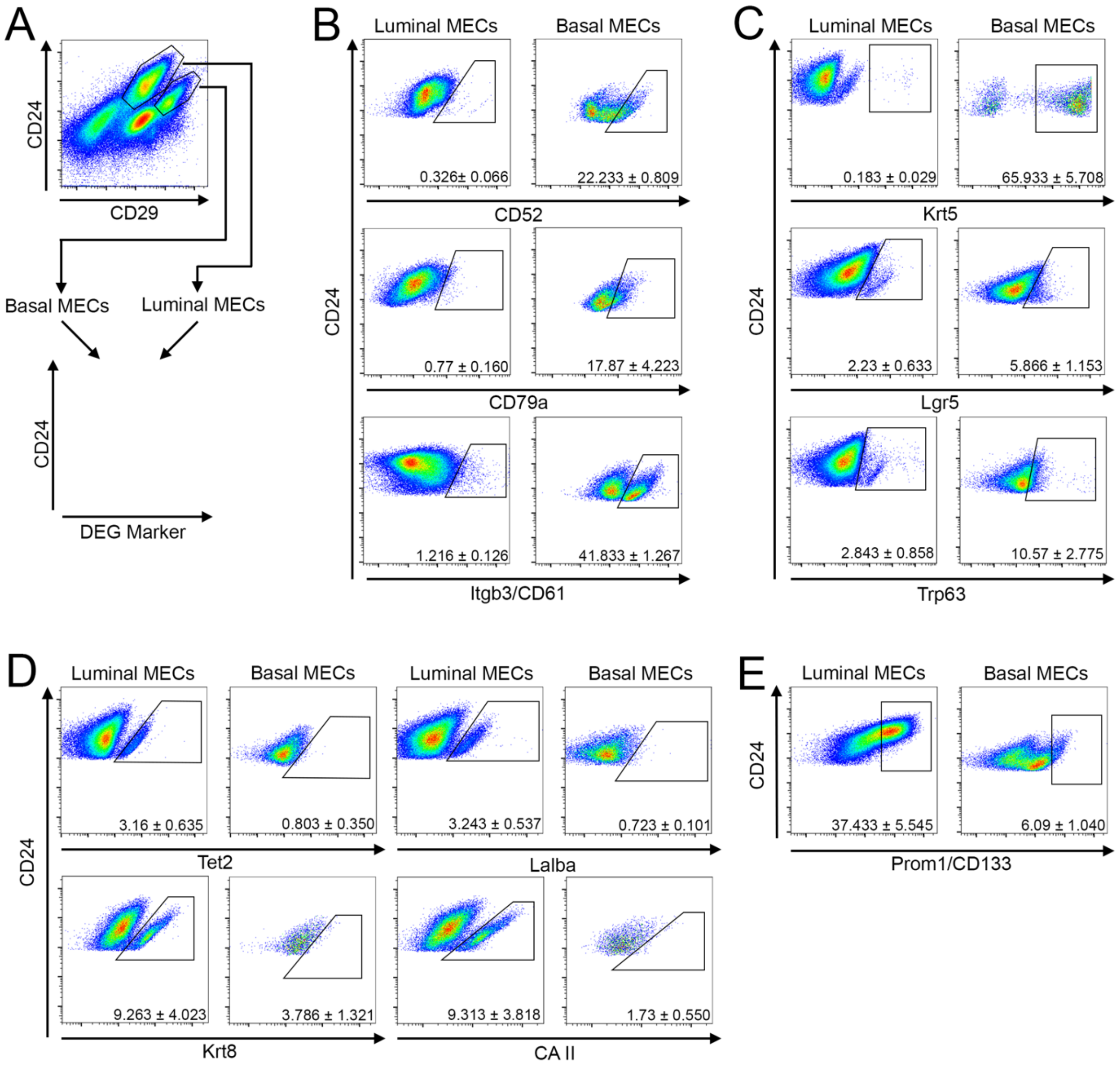

Supplementary Fig. S4

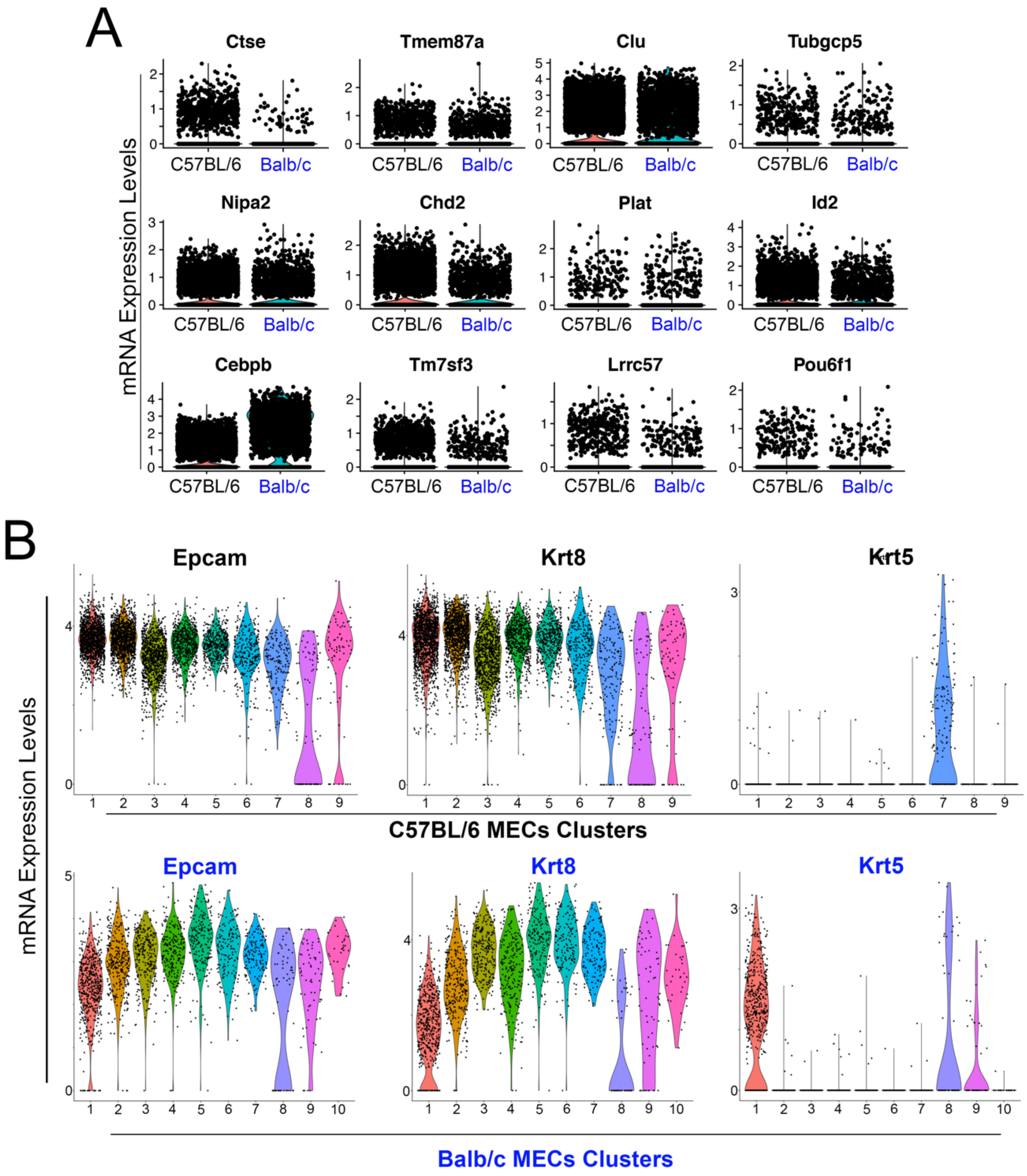

Supplementary Fig. S5

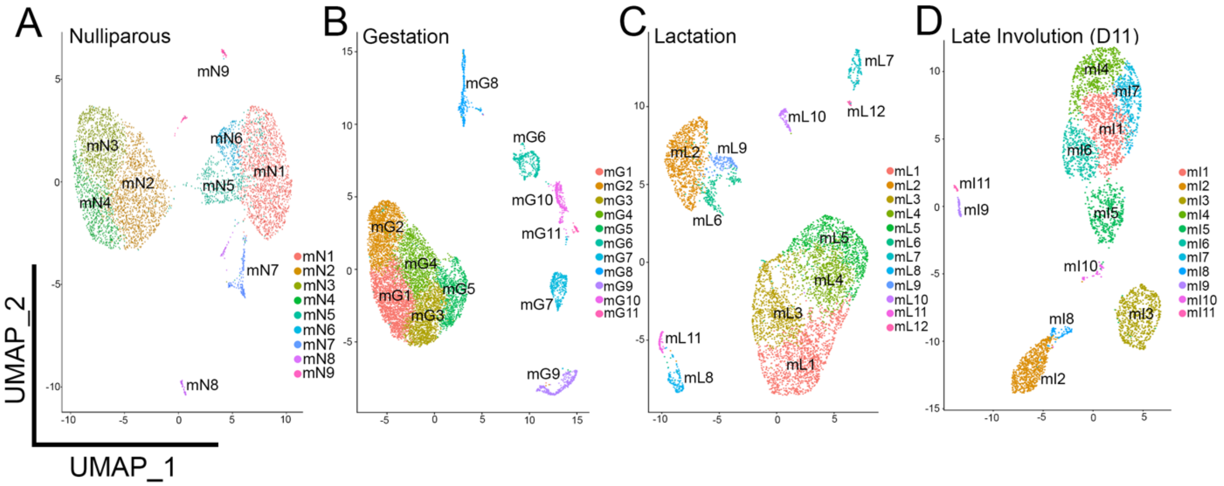

Supplementary Fig. S6

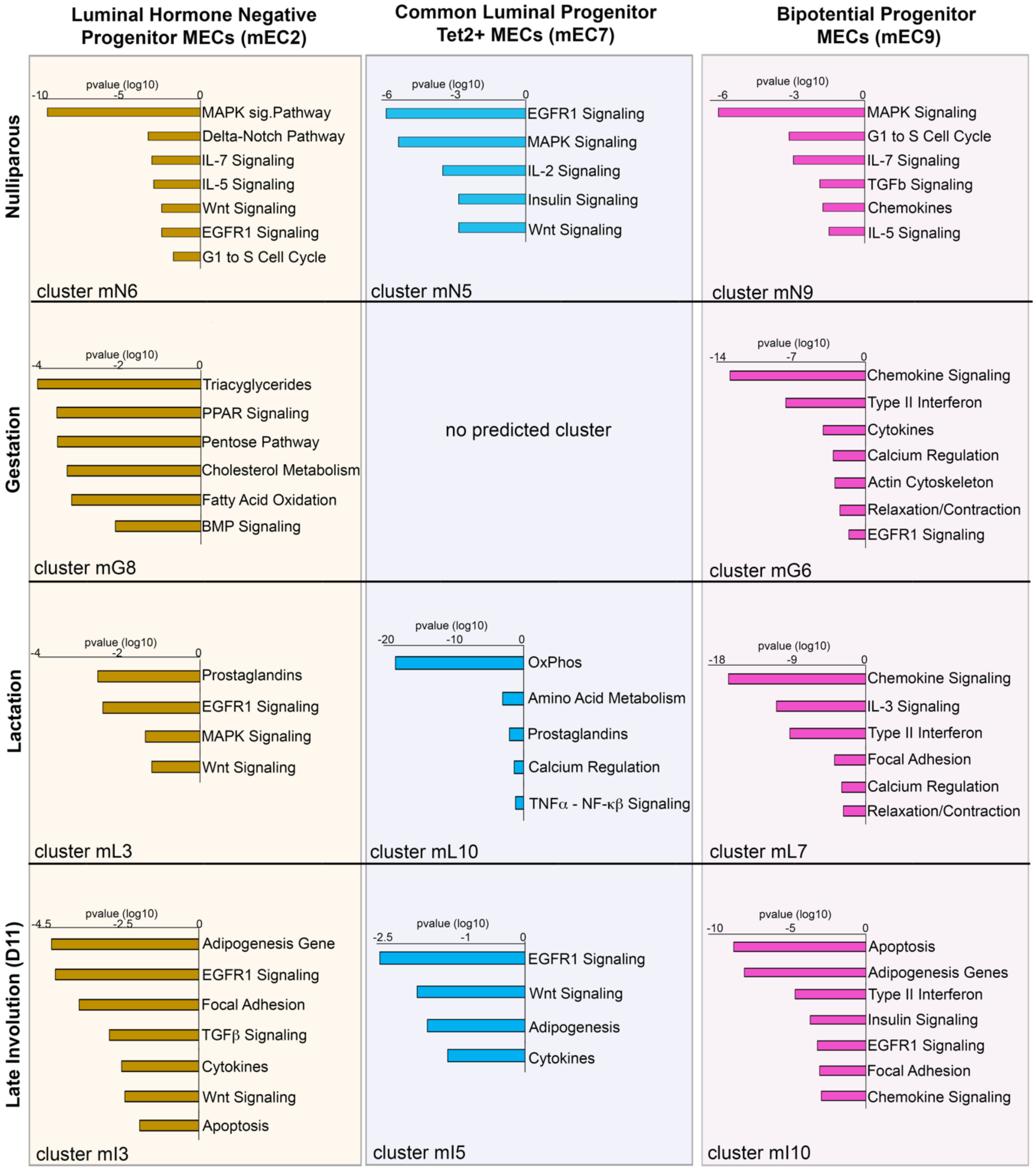

Supplementary Fig. S7

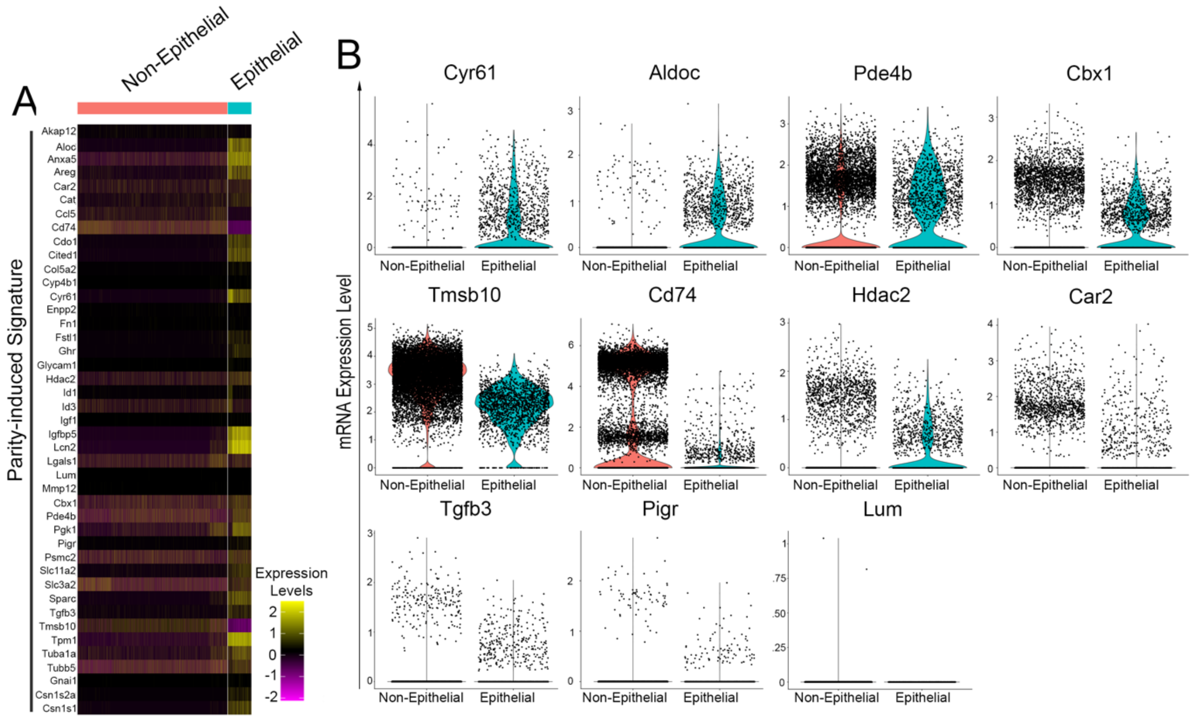

Supplementary Fig. S8

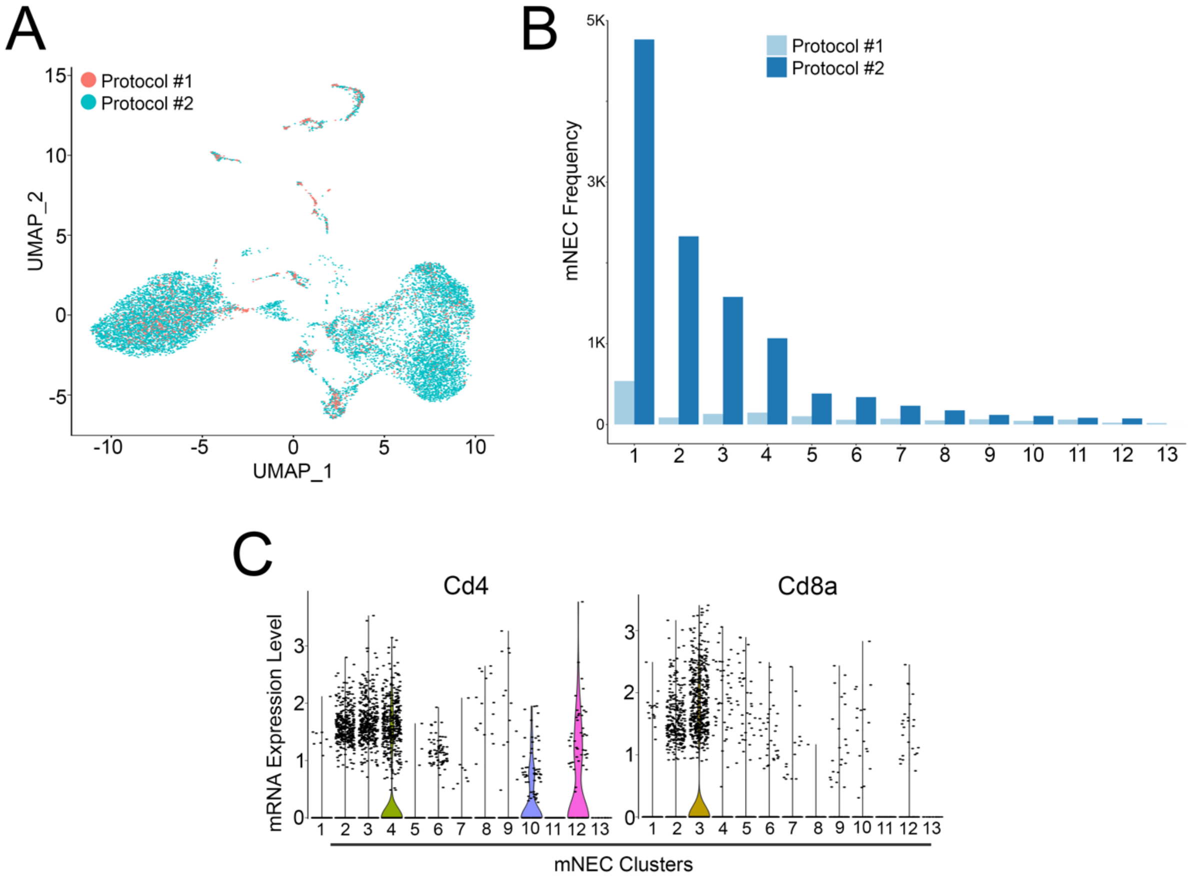

D

Non-Epithelial Cluster	Non-Epithelial Cell Lineage	Lineage Markers
mNEC1	B-Cell	Pkig, Cd72, Myo1e, Pou2f2, H3f3a, Smc6, Ralgps2, Ccr6
mNEC2	Cd4+ T-Cell	Fam101b, Ndrg3, Eef1a1, Kif1b
mNEC3	Cd8+ T-Cell	Dapl1, Evl, Txnip
mNEC4	Tregs	Ikzf4, Tox, Tmem65, Lrig1, Gbp3
mNEC5	Cd8+ NKT-like cells	Xcl1, Ctsw, Ncr1, Klrc1, Klrc2, Eomes, Klre1, Fasl, Sytl3, Ctla2b, Frf1, Car2
mNEC6	B-Cell	Cr2, Hvcn1, Snx2, Ahsa1, Swt1
mNEC7	Dendritic	Batf3, H2-M2, Fscn1, Zmynd15, Tbc1d4, Arhgap31, Ccl22, Cacnb3, Ogfrl1, Pfkfb3, Lrrk1, Il4i1
mNEC8	Cd4+ NKT-like cells	Calca, Trdc, Pxdc1, Zbtb16, Hlf, Sdc1, Smox, Lmo4, Tiam1, F2r, Ikzf3
mNEC9	Macrophage-like cells	Il1rn, Lyz2, Cd14, Clec4d, Emilin2, Tgfbi, Gatm, Havcr2, Lst1, Hmox1, Plbd1, Hexb
mNEC10	Proliferating Treg	Top2a, Mki67, Birc5, Ube2c, Mydgf, Ewsr1, Btf3
mNEC11	Fibroblast	Cxcl1, Ccl7, Igfbp7, Il6, Ccl11, Sparc, Cryab, Procr, Acta2, Dcn, Timp3, Vcam1, Mylk, Pdgfa
mNEC12	NK Cells	Siglech, Ly6c2, Ccr9, Klk1b27, Cd209d, Havcr1, Lefty1, Ly86
mNEC13	Adipocyte-Like cells	Car3, Fabp4, Cfd, Retn, Thrsp, Cdo1, Cidec, Adipog, Pck1, Adig, Nnmt, Apoc1, Cd36, Fcor, Adhfe1, Ppp1r1a, Cavin1, Acadvl

Supplementary Fig. S9

A

Individual	Year of Birth	Ethnicity	Parity
#1	1995	White	Nulliparous
#2	1967	White	Nulliparous
#3	N/A	White	Nulliparous
#4	1994	White	Nulliparous
#5	1984	White	Nulliparous

B

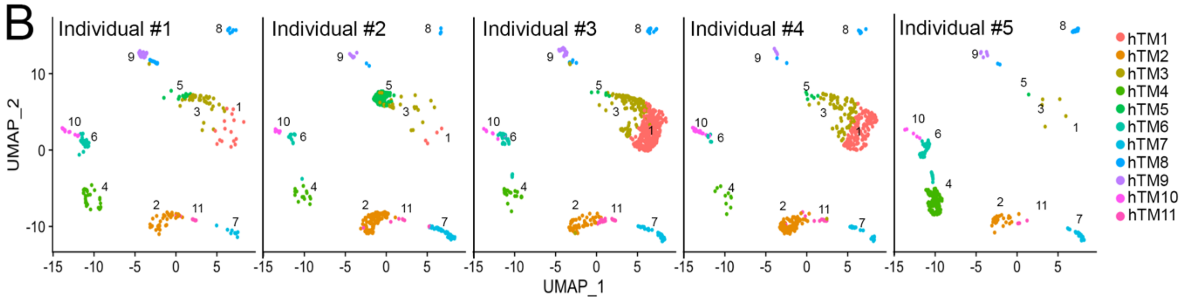

C

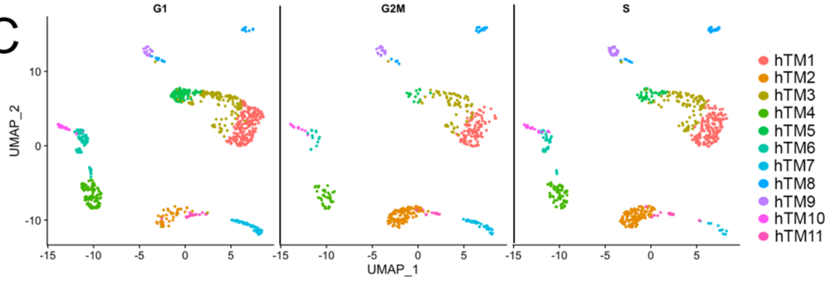

D

	DICE	HPCA	Monaco	NoverHem
hTM1	Monocytes	Fibroblasts	Monocytes	HSCs
hTM2	CD4+ T Cells	T Cells	T Cells	CD8+ T Cells
hTM3	Monocytes	Fibroblasts	Monocytes	HSCs
hTM4	Monocytes	Epithelial Cells	Dendritic Cells	HSCs
hTM5	Monocytes	Fibroblasts	Monocytes	HSCs
hTM6	Monocytes	Epithelial Cells	Dendritic Cells	HSCs
hTM7	Monocytes	Monocytes	Monocytes	Dendritic Cells
hTM8	Monocytes	Tissue Stem Cells	Dendritic Cells	HSCs
hTM9	Monocytes	Endothelial Cells	Monocytes, Progenitors	Monocytes
hTM10	Monocytes	Epithelial Cells	Monocytes	CMPs
hTM11	B Cells	B Cells	B Cells	B Cells

E

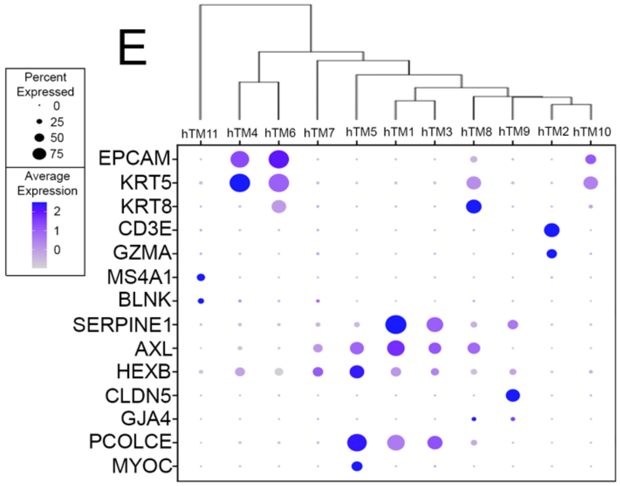

F

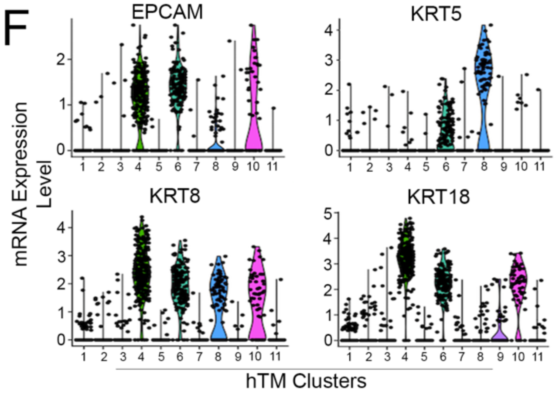

Supplementary Fig. S10

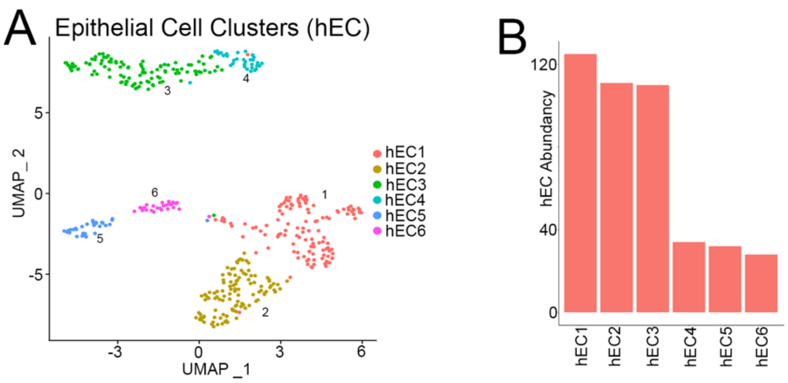

C

Epithelial Clusters	Epithelial Cell Lineage	Lineage-associated genes
hEC1	Differentiated PRLR high Luminal Cells	Krt8, Ankrd30a, Dnajc12, Tmc5, Tbx3, Aff3, Tmem45b, Efhd1, Sytl2, Sfmbt2, Tspan5, Prlr, Galnt6, Gstm3, Pthlh, Itgav, Ereg, Erbb2, Areg, Ar, Fbp1, Krt18, Mlph, Foxa1, Elovl5, Cd164, Muc1, Runx1, Kdm5b, Krt19, Gata3, Cldn4, Cldn7, Itga2, Itgb6
hEC2	Differentiated ER1 high Luminal Cells	Serpina1, Npy1r, Gfra1, Esr1, Piezo2, Nek10, Prex1, Pgr, Cited1, Tspan13, Fbp1, Ar, Foxp1, Krt18, Mlph, Foxa1, Elovl5, Cd164, Muc1, Runx1, Kdm5b, Itgav, Gata3, Cldn4, Armcx3, Cldn7, Itga6
hEC3	Bipotential Progenitor	Gabrp, Ptn, Ncald, Bbox1, Rgs2, Kit, Muc15, Anpep, Prom1, Krt16, Nfatc2, S100a6, Ly6e, Cd14, Krt19, Cldn4, Cldn7, Krt15, Igf2bp2, Slpi, Chi3l1, Lif, Pigr, Elf5, Aldh1a3, Sox9, Fgfbp1, Klf5, Cryab, Anxa1, Folr1, Saa1, Naca, Fth1, Gas5, Itgb6, Krt7, Mmp7, Lamb3
hEC4	Luminal Progenitor PRLR high	Cyp24a1, Ccl20, Slc6a14, Cxcl5, Cxcl1, Saa2, Tnip3, Calml5, Krt15, Igf2bp2, Slpi, Chi3l1, Lif, Pigr, Aldh1a3, Fgfbp1, Anxa1, Elf5, Folr1, Saa1, Cav1, Tpt1, Gnb2l1, Naca, Fth1, Itgb6, Krt7, Itga6, Mmp7, Myo1e
hEC5	Myoepithelial Progenitor	Cxcl14, Tp63, Egr2, Fbxo32, Has3, Ano1, Etv1cldn11, Brd2, Per2, Mme, Actg2, Mylk, Acta2, Dkk3, Glt8d2, Krt17, Krt5, Krt14, Tagln, Myh11, Lamb3, Oxtr, Znf503, Apoe, Stmn1, Itgb4 , Cited2
hEC6	Myoepithelial Differentiated	Wif1, Grp, Cd200, Ntf3, Clmp, Actg2, Krt17, Trnp1, Ptprz1, Ngfr, Lgr4, Krt14, Krt5, Mylk, Acta2, Erg, Dkk3, Glt8d2, Lmod1, Itgb1, Apoe, Sema5a, Spry2, Myo1e, Cav1, Tagln, Lamb3, Znf503, Itgb4, Oxtr

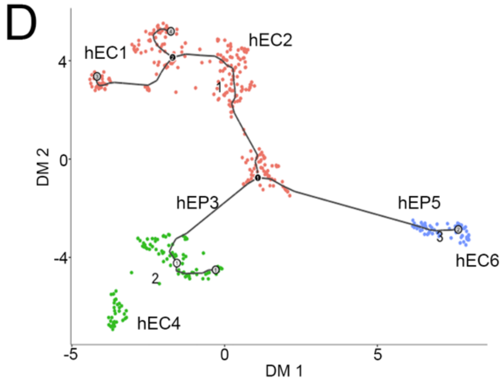

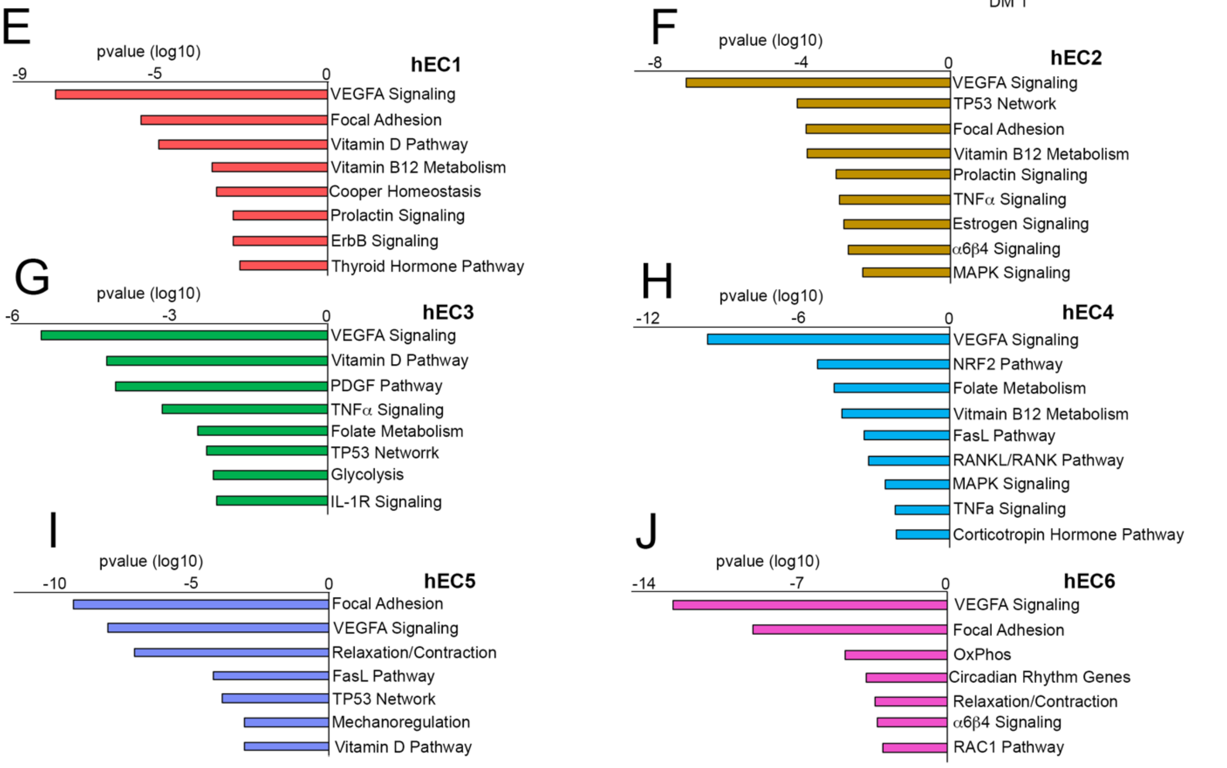

Supplementary Fig. S11

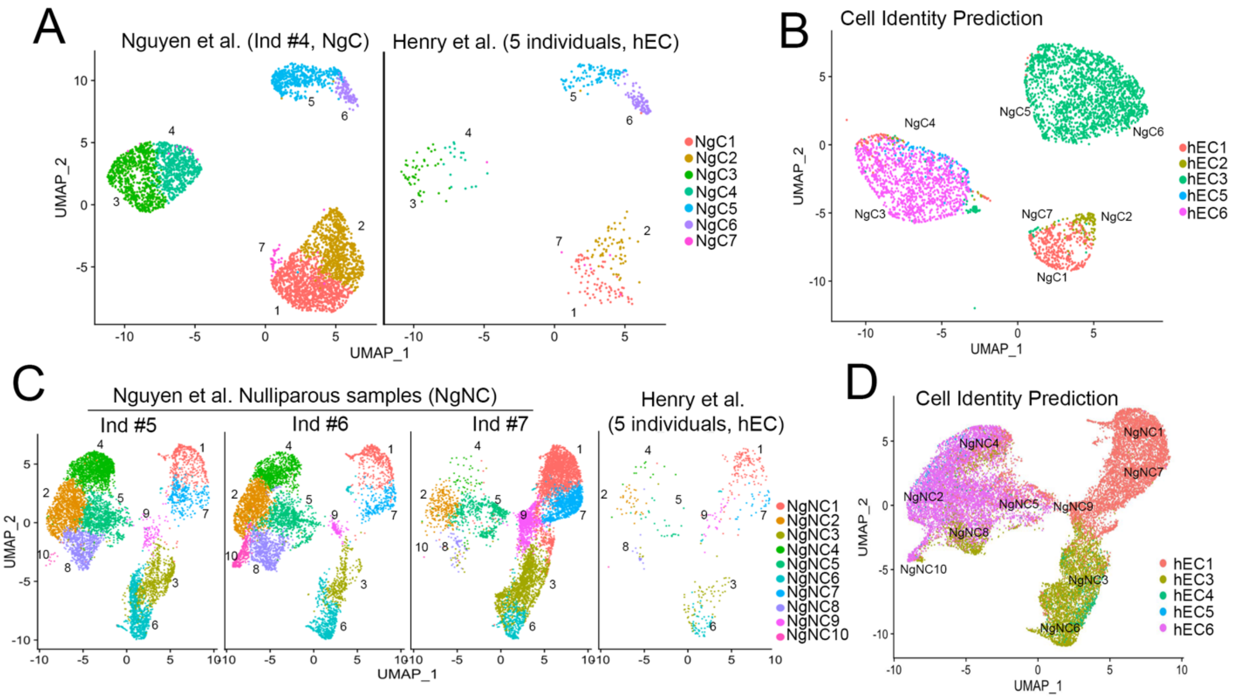

Supplementary Fig. S12

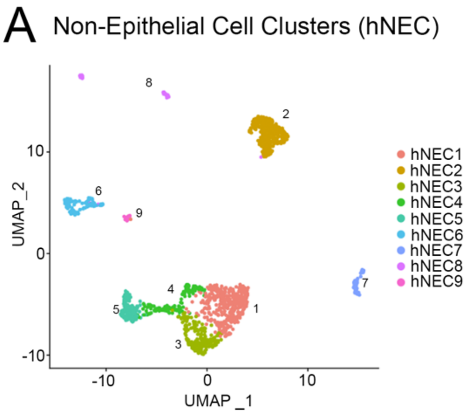

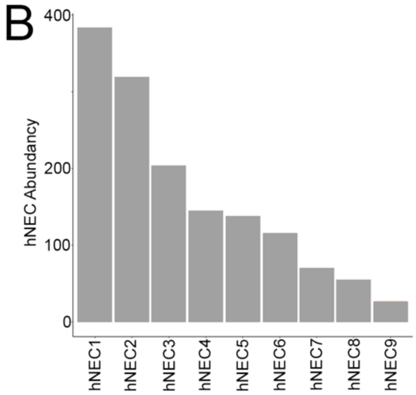

C

Non-Epithelial Clusters	Non-Epithelial Cell Lineage	Lineage-associated genes
hNEC1	Neutrophils	Cxcl1, Efnb2, Frmd4a, Cxcl6, Axl, Trim47, Gpm6b, Mltk, Tnfaip6, Ptgds, Ctsl, Tnn, Col15a1, Serpine1, Sema6a, Igfbp2, Cebpb, Btg3, Cxcl2, Mki67ip, Ldlr, F2r, Pdpn, Apoe, Gas7, Acvrl1, Cd59, Egfr, Icam1, Itgav, Gprc5a, Gfpt2, Il1r1, Bdkrb1, Lif, Cald1, Cd63, Ahcyl1, Pdgfrl, Nfx1
hNEC2	CD8+ NKT-like	Ptprc, Trbc2, Sytl3, Ccl5, Cd3e, Cd3g, Cst7, Cd3d, Cd96, Nkg7, Cd8a, Stat4, Il7r, Klrb1, Gzmk, Gzmm, Gzma, Il2rb, Cxcr4, Cd69, Srgn, Gimap7, Runx3, B2m, Il2rg, Fkbp5, Ddx46
hNEC3	Macrophages	Akr1c1, Lrrc8b, Nptx1, Slc12a8, Creb5, Csf1, Leprel1, Grem2, Msx1, Apoc1, Tnfaip6, Ctsl, Serpine1, Sema6a, Cebpb, Cxcl2, Pdpn, Apoe, Egfr, Itgav, Gprc5a, Gfpt2, Ogn, Cpxm1, Il1r1, Bdkrb1, Lif, Il6, Pparg, Col6a2, Fbln1, Cd63, Ahcyl1, Vim, Plk2, Dcn, Pdgfrl, Itih5, Cpxm1
hNEC4	Monocytes	Arhgap28, Cmbl, Myh10, Fbln7, Prrg3, Hmcn1, Mmp16, Alx4, Irs2, Plk2, Cebpb, Dcn, Hspa1b, Pdgfrl, Itih5, Cpxm1, Lox, Npc2, Ier5l, Klf10, Nr2f1, Sertad1, Igfbp2, Ptgds, Apoe
hNEC5	Adipocytes	Adh1b, Wisp2, Pcolce2, Myoc, Pla2g2a, Fbln2, Lepr, Itgbl1, Negr1, Svep1, Gdf10, Nedd8, Vap, Col15a1, Acvrl1, Egfr, Ogn, Tcf7l2, Klf4, Col6a2, Tspan4, Fbln1, Cd151, Cxcl12, Il33, Cald1, Cd63, Cfd, Cpne3, Itih5, Lox
hNEC6	Dendritic	Aif1, Ms4a6a, Fcer1g, Ms4a7, Itgax, Clec7a, Tyrobp, Il1b, Fcgr2a, C1qc, Cd4, Traf1, Il18, Havcr2, Pla2g7, Batf3, Icam1, Srgn, Runx3, Fkbp5, Cd83, Cd14, Npc2, Nabp1
hNEC7	Endothelial	[illegible] Sox17, Stc1, Tie1, C2cd4b, Eltd1, Plvap, Podxl, Cd59, Icam1, Lmo2, Il6, Pparg
hNEC8	B-lymphocytes	Cd79a, Gng7, Mzb1, Derl3, Igkj5, Igha2, Ms4a1, Igkc, Blnk, Igj, Cxcr4, Cd69, Il2rg, Cd83, Nfx1
hNEC9	Fibroblasts	Ppp1r14a, Gja4, Casq2, Cdh6, Cox4i2, Mylk, Pln, Itga7, Adra2a, Mltk, Cd14, Itih5, Ier5l, Sertad1, Batf3, Il6, Pparg, Nfx1, Nabp1

Supplementary Fig. S13

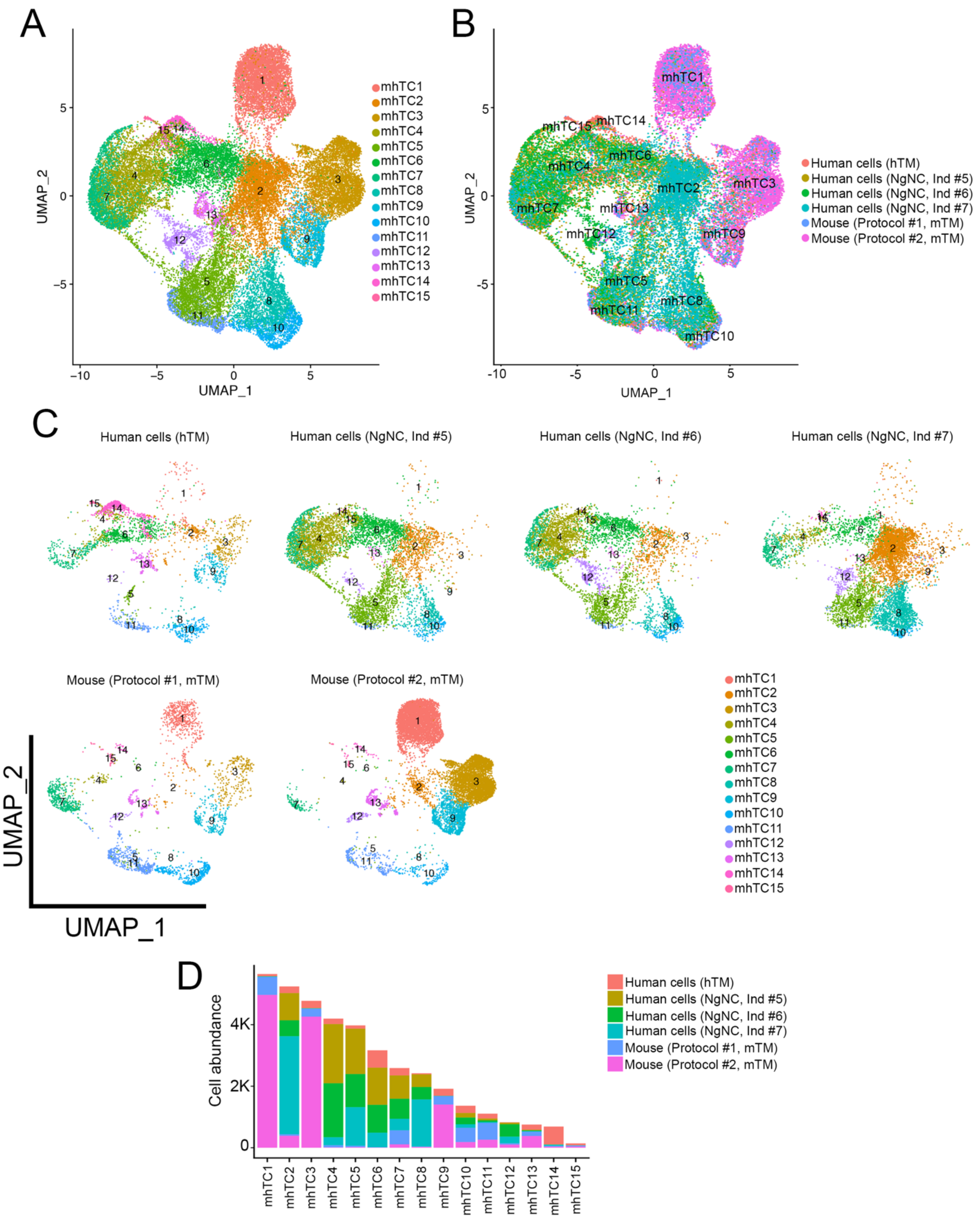

Supplementary Fig. S14

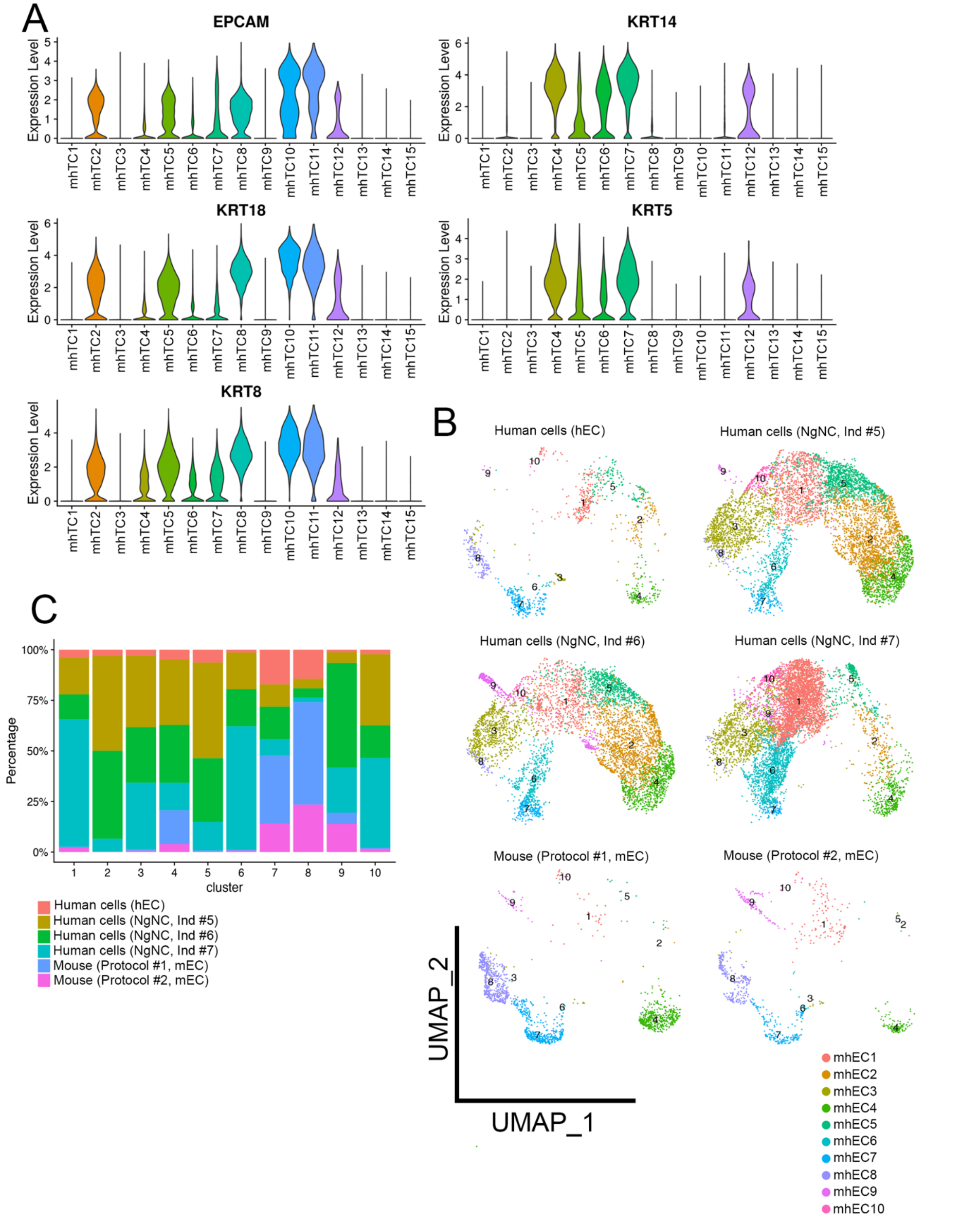

Supplementary Fig. S15
